# Food consumption in the Canary Islands: nutritional implications of food imports and local production

**DOI:** 10.1186/s12889-022-12805-w

**Published:** 2022-02-27

**Authors:** Dirk Godenau, Gloria Martin-Rodriguez, Jose Ignacio Gonzalez-Gomez, Jose Juan Caceres-Hernandez

**Affiliations:** Facultad de Economía, Empresa y Turismo. Campus Guajara, Universidad de La Laguna, 38200 La Laguna, Spain

**Keywords:** Food consumption, Local food production, Food imports, Nutrition, Canary Islands

## Abstract

**Background:**

The composition of the average diet for the Canary Islands’ population has been the subject of concern for the region’s authorities and nutrition specialists. In this study, the composition of the average diet in the Canary Islands is estimated.

**Method:**

The approach is based on secondary data on local production and external trade. The breakdown of the total volume of apparent food consumption into specific product categories marketed to the consumers is achieved by applying hypotheses about losses in the distribution process. The estimation of food intake is obtained by making assumptions about the rates of food wastage in the final stage of consumption. This consumption is expressed not only in terms of edible weight and the market value associated with different food groups according to their local or imported origin, but also in terms of their energy and nutrient content.

**Results:**

The results obtained suggest a high-calorie diet, close to three thousand kilocalories per person per day, with an average cost of around eight euros per person per day. Imported products, with a lower average cost per unit of energy, provide most of the carbohydrates and fats.

**Conclusion:**

This study provides a complementary approach to survey-based evidence and also offers the possibility of evaluating the contributions of local or imported food to the diet.

**Supplementary Information:**

The online version contains supplementary material available at 10.1186/s12889-022-12805-w.

## Background

The basic function of food is nutrition, and therefore food consumption has obvious links to a population’s health. Indeed, this is precisely the case for the Canary Islands’ population, which suffers from serious problems of obesity and, in general, diseases associated with diet [1]. Owing to this, the nutritional characteristics of the population’s diet have been the subject of concern for the region’s authorities and nutrition specialists. These concerns has been incorporated into the specific objective of the Canary Islands Nutritional Survey (ENCA 1997–1998) [2] and forms part of the health objectives integrated into the CDC study (Cardiovascular, Diabetes, Cancer) of the Canary Islands [3]. The results of these and other studies can be consulted in various scientific works [4–8].

Generally, diet composition is estimated from answers provided by individuals to questionnaires asking them to recall the amount and frequency with which they consume certain types of food. These questionnaires are usually validated and applied to representative samples, but require the application of assumptions about standard portions of the foods or dishes declared by respondents, and the results are conditioned by the accuracy of respondents’ statements about their actual consumption.

This paper presents a complementary approach to the composition of the average diet in the Canary Islands from the estimation of food supply that is acquired and finally ingested by consumers, including residents and tourists. It is therefore an alternative approach to the usual ones. This approach estimates the losses in production and distribution processes until the acquisition by consumers at the point of sale, as well as approximating the percentage of acquired production that is wasted and not ultimately ingested by individuals (food waste in households and the in-premises channels). The resulting average diet may provide indications regarding the hypercaloric content of food intake that would help explain the prevalence of certain diseases. However, this approach does not reveal differences in diet composition for different individuals. Thus, the aim of this study is to determine final food consumption, as well as to approximate the percentage of production that is wasted and not finally ingested by individuals.

Diet composition for different population groups is the result of multiple factors such as age or educational level [9, 10], but it is also influenced by food prices and consumer purchasing power. In addition to demand factors such as consumer preferences and budget restrictions [1], other aspects such as the breadth and depth of food supply (including the distribution system) and the regulatory framework affecting product standardization (food composition), relative food prices (support for local production or imports) and consumer perceptions (promotional or awareness campaigns) also play a role [11–17]. In particular, certain elements of regional agri-food policy, such as Régimen Específico de Abastecimiento (Specific Supply Regime, SSR) that aims to make imported food cheaper by offsetting transport costs, or programmes to support crop production aimed at foreign markets, have a notable impact on food supply and demand.^1^ In this sense, the contribution to the diet of local or imported production has an impact on the food security of the Canary Islands’ population in terms of the availability of different food types and the economic capacity to acquire them by different population segments [18].

Proximity foods can have comparative advantage in the case of fresh perishable products, while in the case of imported products, characteristics such as energy concentration and the greater durability of processed products act as mechanisms to overcome greater distances and reduce transportation costs [19]. Therefore, the relative weight of processed foods in a population’s diet can have an impact on the final composition of the diet in terms of the relative weights of certain food groups and introduce distortions regarding the recommendations for a healthy diet, though processed foods can also mean access to a more or less varied and affordable food basket.

In this sense, the aim of this study is to approximate the composition of the diet in the Canary Islands in terms of food groups according to the origin of production. To this end, an approach is adopted that aims to approximate the average apparent consumption for individuals in this population from official data on local production and external trade for food products and food groups. Food intake is measured in terms of edible weight, economic value and energy content, as well as in terms of the main nutrients, i.e., proteins, fats, carbohydrates and alcohol.

## Methods

This study does not aim to explain the determining factors of food consumption in the Canary Islands, nor does it seek to defend that locally produced foods are necessarily more (or less) healthy than imported ones. The study design is descriptive, observational and retrospective. As mentioned above, regional food consumption has been estimated by approximating apparent consumption according to official statistics on local production and external trade. This approximation to Net Food Consumption will henceforth be denoted as NFC. Although a detailed explanation of the assumptions introduced for the calculation of local or imported production of different foods can be found in [16], some of the basic elements for understanding the nature of the procedures are briefly described below, and greater attention is paid to those aspects that refer to the measurement of the dimensions considered and the assessment of intake based on assumptions about the amount of food waste.

### Estimating local and imported food supply

Local production that is exported and imported production that is re-exported do not contribute to satisfying regional consumption and are therefore eliminated in the calculation of the production consumed by the Canary Islands’ population. In addition, some food production may be used as an input for obtaining processed products and, in that case, has not been counted in the estimate of consumption of unprocessed food, but, where appropriate, contributes to the production of the food industry, which is also recorded in official statistics. Approximations of food consumption have been obtained for the following four groups: unprocessed plant products, unprocessed livestock products, unprocessed fishery products and food industry products. The calculation of the net production of local or imported origin marketed in the local market for a particular foodstuff, *Q*^*L*^ and *Q*^*I*^, gives the apparent consumption of that foodstuff, while the aggregation of the respective local and imported production results in apparent consumption for the food group defined by the aggregation in question. In formal terms, the consumption of a food or food group can be defined as *C* = *Q*^*L*^ + *Q*^*I*^, where local or imported production corresponds to the food or food group under consideration, expressed in edible weight (grams per person per day), market value at average retail prices (euros per person per day), energy content (kilocalories per person per day), or protein, fat, carbohydrate, alcohol and saturated fatty acid content (grams per person per day). Finally, and in order to avoid distortions arising from the storage of production that has not been consumed, it has been decided to use five-year averages of local or imported production consumed per person per day for each of the years of the five-year period, weighted by population and number of days in the year in question. This population includes both the resident and tourist populations (in terms of population equivalent, tourists represent 12% of the total population). Tourist consumption is impossible to isolate by approaching consumption through information on production and trade. In fact, it adds an element of heterogeneity, because the tourist eating habits during their holiday may differ from their usual pattern. Moreover, this usual pattern in their places of origin may also differ from the habits of the Canary Islands’ population [20, 21].

The definition of foods for which it is possible to approximate consumption is conditioned by the availability of information on local production and external trade. Trade statistics with an 8-digit breakdown of TARIC codes have been used. This implies that some food groups may be quite heterogeneous, particularly in the case of food industry. In these cases, the transformation of trade weights into edible weights or energy content requires the use of conversion factors that can only be considered an approximation to reality.

Another issue to be taken into account when interpreting the results is their dependence on the definition of local production. In the case of unprocessed products, the place of production identifies their origin. However, in the case of the food industry, many foodstuffs are obtained as a final product in the Canary Islands from simple transformations of the imported product, such as the addition of water or packaging. Therefore, in addition to the criterion of the National Accounts, which identifies local production in accordance with the location of the producing company, a more restrictive criterion has also been considered, which identifies as production of the local food industry only that which is obtained from unprocessed foodstuffs of basically local origin. In practice, this definition of pure local industry has been limited to the production of cheese, wine, bottled water and olive oil.

### Conversion of food into energy and nutrients

The conversion of amounts consumed into energy and nutrients has been done as follows. The BEDCA database (Spanish Food Composition Database) records the kilocalorie content per 100 g of edible portion of the foods considered. Once production volumes have been converted into edible weight as explained in [16], it is possible to evaluate their energy content. In this sense, the nutritional equivalents (kilocalories, grams of protein, grams of fat, grams of carbohydrates, grams of alcohol, and grams of saturated fatty acids, per 100 g of edible portion) published in BEDCA and some records included in [22] have been applied. For those specific foods for which no information was available in these databases, the nutritional equivalents corresponding to another similar product in the same group have been chosen.

In the case of meat and fish, once the production volumes have been converted into equivalent edible weight of fresh product, it is possible to evaluate the energy content from the nutritional equivalents (kilocalories, grams of protein, fat, carbohydrates and of alcohol per 100 g of edible portion) published in BEDCA for fresh products. However, for some products not covered by the database, the energy equivalents indicated in FAO reports have been used. For processed foods for which no information was available in these databases, nutritional equivalents published by other sources or indicated on the commercial labels of some products of the type concerned have been used. The conversion factors finally used are shown in Additional file 1.

The amounts of protein, fat, carbohydrate and alcohol of local or imported products were converted into their caloric equivalent by means of the ratios 4, 9, 4 and 7 kcal per gram, respectively. These caloric equivalents approximately coincide with the averages in kilocalories estimated directly from the nutritional information for the foods considered in each group.

### Estimating food intake

For the purpose of estimating consumption, food production is assessed at the point of purchase by the final consumer of the product, so that, if intake is to be approximated, food waste at the final stage of consumption must be estimated. In formal terms, the intake of a food or food group can be defined as *D* = *Q*^*L*, *D*^ + *Q*^*I*, *D*^, where *Q*^*L*, *D*^ = (1 − *w*)*Q*^*L*^ and *Q*^*I*, *D*^ = (1 − *w*)*Q*^*I*^, where *w* is the waste rate, which is considered equal for local or imported production within the same food group.

As the recent FAO report [23] on global food waste shows, there is a great diversity of waste rates according to countries and types of food. It would therefore be appropriate to use specific information for the Canary Islands, which is not available, however. The existing information for Spain, Europe or even other territories also has empirical and methodological limitations for use in the context of this study. For these reasons, it has been necessary to apply the food waste rates by food group included in Additional file 2.

The application of these food waste rates implies a total volume of food waste of almost 97 kg, or more than 98 kg of commercial weight per person per year in the five-year periods 2012–16 or 2013–17, respectively. This places total waste at levels close to those of other European countries and well above household waste calculations according to 2017 data in the Panel for the Quantification of Food Waste in Spanish Households, prepared by the Ministry of Agriculture, Fisheries, Food and the Environment of the Government of Spain. Volume of avoidable food waste in Spanish households is estimated at 116 kg per person per year, while the European Union average waste stands at 119 kg per person per year [24]. Also for the European Union, [25–27] estimate a volume of avoidable food waste in households of 76, 97 and 92 kg per person per year, respectively, while if food service waste (restaurants, catering, etc.) is added, these figures would rise to 101 and 113 kg according to [25, 27], respectively. Other studies put the volume of avoidable waste in households in Denmark and the United Kingdom, respectively, at 103 and 85 kg per person per year [28, 29].

The calculations have been made from records corresponding to the period 2012–2017. A multi-sourced database was constructed, and the results have been obtained using spreadsheet software (Microsoft Excel). No special approval or agreement was needed to start the study.

## Results

This section describes the results of applying the criteria set out in the previous methodological section (NFC approach). Section 3.1 explains the composition of the diet obtained in the different dimensions and the food groups indicated in Additional file 1. Some results are also included on the nutritional intensity per unit of edible weight and of market value that may influence consumption decisions and the composition of the food shopping basket. In Section 3.2, the approximations of the intake in grams of edible weight in each one of the four food groups commented above, are also expressed in terms of energy content, nutrients and market value according to product origins. Although the calculations have been made for the five-year periods 2012–2016 and 2013–2017, the stability of the results makes it advisable, for reasons of space, to include only those corresponding to the second of these five-year periods. However, the disaggregated results of both five-year periods can be found in Additional file 3.

### Diet composition according to food groups

Considering the estimate of the production actually consumed (ingested) once food waste is eliminated in the final stage of consumption, Table 1 shows the composition of the resulting diet for the Canary Islands’ population in terms of the market value, edible weight and nutrient contents of different food groups. Its interpretation requires taking into account that the products of the food industry represent about 70% of the edible weight, 75% of the caloric contribution and more than 60% of the market value. The estimated solid intake is a little over 1600 g per person per day, while beverages represent just over 1300 g per person per day, highlighting the high consumption of bottled water. The value at retail prices of the purchased production that is transformed into the final intake is close to eight euros per person and day, while the production ingested represents an energy contribution per person and day above three thousand kilocalories. The group of products based on cereals or flour, followed by those on oils and fats, is the main contributor to this energy intake. Other groups included in the food industry, such as dairy products or the meat and fish products, are also very relevant. Oils and fats, as well as sauces and condiments and also preparations of meat and fish products, are the groups that concentrate the fat content, while the group that provides the most carbohydrates is the one based on cereals and flour. As expected, a large part of the proteins are of animal origin (meat, fish, dairy products and eggs account for 65% of the total proteins consumed). It is noteworthy that the group of sugar, cocoa preparations and confectionery provides as much carbohydrate as the consumption of tubers (mainly potatoes) and more than the consumption of fruits.

**Table 1:** Composition of the average diet per person per day in the Canary Islands (NFC)^a^

Products	Value (euros)	Edible weight (kg)	Energy (kcal)	Proteins (g)	Fats (g)	Carbo-hydrates (g)	Alcohol (g)	Saturated fatty acids (g)
Edible tubers	0.194	0.131	96.871	2.853	0.266	20.241	0.000	0.038
Vegetables	0.602	0.206	58.775	3.327	0.608	9.809	0.000	0.127
Legumes	0.014	0.005	16.322	1.207	0.150	2.465	0.000	0.019
Fruits	0.529	0.163	100.956	1.627	2.025	18.196	0.000	0.301
Cereals	0.027	0.023	88.729	1.809	0.427	19.029	0.000	0.093
Meat and edible offal	0.898	0.122	198.065	25.597	10.543	0.053	0.000	3.633
Milk	0.126	0.153	99.568	4.687	5.821	7.200	0.000	3.517
Eggs	0.105	0.028	42.283	3.524	3.129	0.000	0.000	0.872
Honey	0.013	0.001	4.721	0.007	0.000	1.151	0.000	0.000
Fish	0.275	0.031	33.351	5.483	1.204	0.116	0.000	0.277
Crustaceans	0.119	0.010	9.003	1.882	0.112	0.083	0.000	0.019
Molluscs	0.101	0.009	7.441	1.476	0.127	0.082	0.000	0.033
Other aquatic invertebrates	0.001	0.000	0.002	0.002	0.001	0.000	0.000	0.000
Algae	0.000	0.000	0.002	0.000	0.000	0.000	0.000	0.000
Flour	0.011	0.010	33.264	1.000	0.011	7.118	0.000	0.002
Prep. of cereals or flour^b^	0.629	0.187	525.756	15.951	9.280	93.077	0.000	3.681
Prep. of vegetables or fruits^b^	0.466	0.127	83.199	1.306	0.943	17.445	0.000	0.442
Meat or fisheries prep.^b^	0.681	0.091	269.955	17.927	21.227	1.864	0.000	7.795
Soups and brouths	0.040	0.005	15.682	0.593	0.153	2.921	0.000	0.061
Dairy products	0.962	0.172	311.339	12.162	18.789	23.245	0.000	3.215
Ice cream^b^	0.122	0.020	41.730	0.771	2.136	4.826	0.000	1.441
Eggs not in shell^b^	0.008	0.002	14.616	0.698	1.305	0.046	0.000	0.396
Oils and fats	0.098	0.045	379.111	0.121	42.579	0.121	0.000	6.329
Coffee, teas and extracts^b^	0.107	0.000	0.308	0.014	0.012	0.012	0.000	0.001
Sugar and cocoa prep.^b^	0.268	0.032	130.291	1.786	4.980	20.347	0.000	1.051
Sauces and condiments^b^	0.086	0.032	193.383	0.618	21.283	0.454	0.000	3.116
Other food preparations	0.079	0.015	16.587	3.694	0.168	0.000	0.000	0.037
Bottled water	0.328	0.862	0.000	0.000	0.000	0.000	0.000	0.000
Non-alcoholic beverages	0.103	0.163	71.670	0.000	0.000	17.510	0.000	0.000
Alcoholic beverages	0.933	0.308	195.924	1.168	0.000	7.407	23.183	0.000
Food products	7.923	2954	3038.905	111.292	147.277	274.820	23.183	36.497

^a^ Estimates are made of the production ingested once food waste has been eliminated in the final stage of consumption

^b^ Complete definitions of these food groups in Additional file 3

In foods based on cereals or flour, the energy contribution is mainly due to the carbohydrate content, while the oil and fat group contributes almost exclusively due to its fat content, which is also relevant in dairy products and meat or fish preparations. The participation in terms of edible weight of food groups with high energy density finally produces a hypercaloric diet typical of industrialized countries. According to the joint WHO and FAO report ([30], p. 15), the average calorie supply estimated for these countries from their agri-food balances was 3440 kcal per person per day in 2015. According to our calculations, the caloric equivalent for the average production acquired by an inhabitant of the Canary Islands would also be close to this figure (3390 kcal per day), while even if food waste is eliminated in the final stage of consumption, the average caloric intake exceeds three thousand kilocalories (3039 kcal, as indicated in Table 1).

From the results in Table 1, the differences in the energy and nutritional intensity of the different food groups per unit of edible weight and per euro spent can be obtained. Specific results for food groups can be derived from the information included in Additional file 3. Unprocessed livestock products and products from the food industry are the most energy-intensive, while processed foods provide the cheapest energy and, conversely, the most expensive energy is from unprocessed fish products (Table 2).

**Table 2:** Average energy and nutritional contribution per euro of market value by food group (NFC)

	Energy (kcal)	Proteins (g)	Fats (g)	Carbohydrates (g)	Alcohol (g)
Primary crops	264.58	7.92	2.54	51.02	0.00
Non processed fisheries	100.56	17.86	2.92	0.57	0.00
Non processed livestock	301.77	29.61	17.07	7.36	0.00
Food industry products	464.05	11.75	24.98	39.92	4.71

Again, unprocessed livestock products and those of the food industry are the highest in fat and also in saturated fatty acids, while the fats contained in processed products are the lowest in unit cost. As regards carbohydrates, unprocessed plant products have a higher content than processed products, both per unit weight and per unit value. And in terms of protein intake, the content per unit weight ingested is higher in unprocessed animal products than in food industry ones. In addition, the proteins obtained from meat are cheaper than those derived from the consumption of fish products.

### Diet composition according to product origin

One of the advantages of the NFC approach is that the contribution to the diet of different food groups can be estimated according to their origin. In the case of the Canary Islands, the differences in the share of local or imported production in edible weight, market value or energy content (see Additional file 3) are a reflection of local productive specialisation. The most important local production in terms of edible weight is bottled water, which, however, has no energy content and little value, which explains why its share in consumption in terms of edible weight is higher than if production is measured in terms of value or kilocalories. Among unprocessed foods, some local products with more or less high shares, such as fruit and, above all, vegetables and tubers, have high unit values compared to their energy density, so that they also help to explain these differences.

In any case, these amounts depend notably on the definition adopted for local food industry. Looking at the food industry product group, it can be seen that the share of locally sourced carbohydrates, which was close to 50% in the scenario defined by the National Accounts (Option A), is almost zero in the alternative option (Option B).^2^ If we take into account the contribution of proteins and fats, the relative shares in the first scenario are above 30% and close to 15%, respectively, and fall in the second scenario to levels close to 5% in both cases. The reduction is even greater in saturated fatty acids. Moreover, in the case of alcohol, this drop also occurs when local production is limited to wines of local origin.

As a summary, Table 3 shows the relative contributions of the groups that contain local and imported food products in each of the dimensions evaluated and under the two scenarios related to the identification of local production (options A and B). The share of processed foods in the edible weight of products ingested and their higher energy intensity per unit of edible weight explain that they represent three quarters of the total caloric intake. Using the conventional definition of local production, the energy density of processed foods of imported origin is more than three times that of local processed foods, so that the share of the former represents more than half of the caloric intake. In this respect, it should also be noted that the large volume of local bottled water significantly reduces the energy density of local foods in this group, especially when those local processed foods are limited to water, wine, oil and cheese.

**Table 3:** Net food intake per person per day according to food group and product origin (NFC)

	Option A (National Accounts)		Option B (Pure local food industry)		Option A (National Accounts)		Option B (Pure local food industry)	
	Local	Imported	Local	Imported	Local	Imported	Local	Imported
	Edible weight (g)				Energy (kcal)			
Primary crops	276.51	252.53	276.51	252.53	137.00	224.66	137.00	224.66
Non processed fisheries	3.98	45.79	3.98	45.79	5.06	44.74	5.06	44.74
Non processed livestock	38.64	266.47	38.64	266.47	57.00	287.64	57.00	287.64
Food industry products	1229.15	841.39	696.86	1373.68	685.19	1597.62	64.88	2217.94
	Proteins (g)				Fats (g)			
Primary crops	4.29	6.53	4.29	6.53	1.53	1.95	1.53	1.95
Non processed fisheries	0.80	8.04	0.80	8.04	0.19	1.25	0.19	1.25
Non processed livestock	5.73	28.09	5.73	28.09	3.57	15.92	3.57	15.92
Food industry products	18.94	38.87	3.20	54.61	17.87	105.00	4.88	117.98
	Saturated fatty acids (g)				Carbohydrates (g)			
Primary crops	0.25	0.33	0.25	0.33	25.56	44.18	25.56	44.18
Non processed fisheries	0.05	0.28	0.05	0.28	0.00	0.28	0.00	0.28
Non processed livestock	1.09	6.93	1.09	6.93	0.47	7.93	0.47	7.93
Food industry products	5.42	22.15	0.00	27.57	96.13	100.26	0.03	196.36
	Alcohol (g)				Value (euros)			
Primary crops	0.00	0.00	0.00	0.00	0.75	0.61	0.75	0.61
Non processed fisheries	0.00	0.00	0.00	0.00	0.09	0.41	0.09	0.41
Non processed livestock	0.00	0.00	0.00	0.00	0.21	0.94	0.21	0.94
Food industry products	8.35	14.83	1.20	21.99	1.80	3.12	0.48	4.44

Products of imported origin are the main contributors to fat in the average intake, whatever the scenario considered for the identification of local production. There is 84% (option A) or 93% (option B) of fats which correspond to imported products. In the case of saturated fats, these percentages reach similar values of 81 and 96%, respectively. However, in terms of carbohydrates, the local food industry contributes significantly only when the conventional criterion of local production is adopted (35%), while its contribution is practically cancelled out when the more restrictive scenario for the local industry is considered. A significant reduction also occurs when examining the change in the protein contribution of the local food industry according to the scenario considered. For all food products, 73% of protein is imported in Option A, compared to 87% in Option B. Something similar happens with the alcohol content of the diet, mainly as a consequence of the different treatment of beer production carried out in the Canary Islands, so that imports contribute 64% of the alcohol ingested in option A and 95% in option B.

**Fig. 1:**
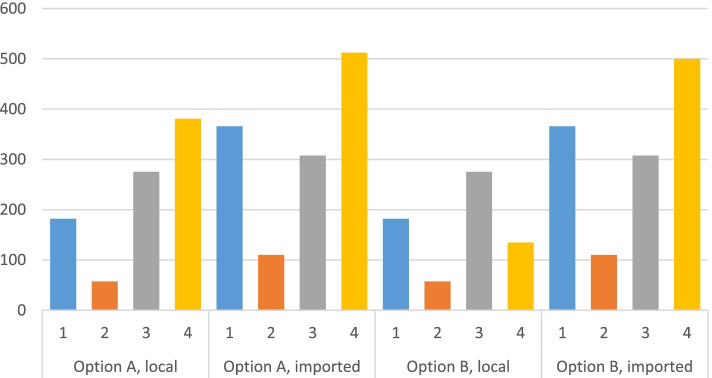
Caloric contribution (kilocalories) per euro according to food group and product origin (NFC). Note: 1. Primary crops; 2. Non processed fisheries; 3. Non processed livestock; 4. Food industry products

Although the unit of edible weight ingested of food industry products is on average cheaper than in any of the other groups, the relative share of processed foods in the diet (more than 2 kg of edible weight ingested per person per day, including water and other beverages) ends up making this the most important group in terms of expenditure per person per day, both for local products (almost 2 euros) and for imported foods (more than 3 euros), at least if local production is identified in conventional National Accounts terms. However, if the more restrictive definition of local production is adopted (option B), the contribution to expenditure from imported processed foodstuffs obviously increases (more than 4 euros), and among the products of local origin the most relevant share is for unprocessed plant products (more than 70 eurocents). On the other hand, as shown in Fig. 1, the caloric contribution per euro spent on imported products is always higher than that corresponding to products from the same group for local origin.

In summary, even in the National Accounts’ scenario, the imported food industry products are those that contribute most to the diet in terms of protein, fat, carbohydrates and alcohol, standing out especially in the section on fats (105 g per person per day out of a total of just under 150 g). Two thirds of the intake of carbohydrates (275 g per person per day) corresponds to the food industry (196 g per person per day), and although the National Accounts criterion suggests a certain balance in the distribution of this production according to origin, in scenario B almost all of these hydrates are attributed to processed foods of imported origin.

Although the information concerning the large food groups is sufficiently revealing in relation to the contribution of certain imported productions, it is also interesting to highlight the incidence of certain specific food groups. Following the conventional criterion of the National Accounts (option A), local production of cereal or flour-based pre-prepared dishes represents the main local caloric contribution (270 kcal per person per day). In the alternative scenario (option B), all this production is considered to be of imported origin, and the main local caloric contribution within the food industry becomes cheese (almost 60 kcal per person per day), with a contribution similar to that of local tubers (61 kcal per person per day). In this second scenario, and within the imported food industry products, pre-prepared dishes based on cereals or flour (526 kcal per person per day) and oils and fats (379 kcal per person per day) stand out. Other significant contributions correspond to the food groups of pre-prepared dishes based on meat and fishery products (270 kcal per person per day) or dairy products (255 kcal per person per day).

## Discussion

This section compares the results obtained with those of studies based on questionnaires [2–4, 7] and highlights the most relevant conclusions. Table 4 shows the composition of the diet in food groups set up in such a way as to allow comparison with the results of the ENCA and CDC studies. Following the NFC approach, solid intake is mainly provided by the group of tubers, pulses, vegetables and fruits, which together provide slightly more than 600 g per person per day, corresponding especially to vegetables, fruits and tubers, in that order (see Additional file 3). Another group of foods with a very relevant weight in the diet is dairy products, which represent more than 300 g per person per day, of which approximately half corresponds to milk consumption. The high contribution of the group of cereals and cereal products is explained by the contribution of cereal and flour-based pre-prepared dishes, which represent almost 200 g per person per day. The combined contribution of meat and fish, including pre-prepared ones, also exceeds 250 g per person per day. With regard to beverages, the main contribution to the group of non-alcoholic beverages corresponds to bottled water, with a consumption of just under one litre per person per day. Furthermore, in alcoholic beverages, the consumption of beer is three times higher than the consumption of wine (see Additional file 3).

**Table 4:** Composition of diet (grams per person and day)

	NFC^a^ 2013–2017	CDC^b^ 2000–2003	ENCA^c^ 1997–1998
Edible tubers, legumes, vegetables and fruits	633.12	915.08	498.50
Cereals and preparations of cereals	219.97	110.97	125.30
Milk, cheese and other dairy products	344.62	609.28	390.70
Eggs	30.39	16.06	25.10
Meat and preparations of meat	167.52	171.45	105.10
Fisheries and preparations of fisheries	95.04	41.94	45.80
Oils and fats	45.31	17.58	27.90
Sugar. cocoa preparations and sugar confectionery	33.64	49.80	81.90
Miscellany	51.76	7.68	14.50
Non alcoholic beverages	1025.15	1375.70	590.50
Alcoholic beverages	307.93	92.21	62.80
Total	2954.46	3407.75	1968.10

^a^ Prepared by authors from official statistics on local production and external trade for the five-year period 2013–2017

^b^ CDC 2000–2003 (compiled by the authors on the basis of (7))

^c^ ENCA 1997–1998 (compiled by the authors on the basis of (4) and (7)

The comparison of this composition with the results obtained in studies such as ENCA and CDC is limited, given that in these two cases the questionnaires are applied to the resident population in certain age groups and, therefore, the population considered is not comparable to the reference population in our study.^3^ In this sense, the differences in the consumption pattern of the resident and tourist population and of different age groups may help explain some of the differences found [20]. In addition, the chronological differences may reflect possible changes in diet over a time horizon of about 20 years. In any case, the three food groups with the greatest weight in solid intake according to these studies coincide with those discussed previously.

**Fig. 2:**
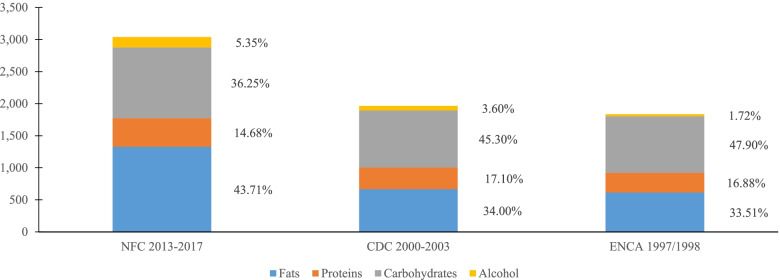
Nutrient contribution to caloric intake (kilocalories per person per day)^(1)^. ^1^ Prepared by authors based on official statistics on local production and external trade for the five-year period 2013–2017 and information obtained from the CDC 2000–2003 study (7) and the ENCA 1997–1998 study (4,7)

The explanations for the differences found in the composition of the diet in terms of edible weight ingested can respond, at least in part, to the fact that a significant part of the consumption collected in the questionnaires corresponds to dishes prepared in households, while records of marketed production compute as individual foods many of which are then used in the preparation of these dishes.^4^ However, one would expect these effects to be mitigated in terms of energy content. Figure 2 shows that both in terms of total caloric intake and even relative contributions of nutrients (protein, carbohydrate, fat and alcohol) to that caloric intake, differences between estimates derived from production and trade records and those from questionnaires persist. According to the NFC approach, the average diet per person per day is 3000 kcal, with fat and carbohydrate contributing slightly more and slightly less than 40%, respectively. However, the results of the ENCA and CDC studies suggest an average caloric intake per person per day of less than 2 thousand kilocalories, of which the basic contribution corresponds to carbohydrates and secondly to fats.

In the overall diet resulting from the application of the NFC approach, proteins, fats, carbohydrates and alcohol contribute, respectively, 15, 44, 36 and 5% of the total caloric intake. In other words, this is a diet in which the distribution between fats and carbohydrates is far from the internationally recommended relative weights ([30], p. 56), due to the excessive relative weight of the former and the lower weight of the latter. It is noted that in the group of fats there is an excessive presence of saturated fats; it should be less than 10%, but in fact, it is 25%. According to these recommendations, in the group of carbohydrates, sugars should account for less than 10% of the total energy intake, but their amounts in the diet estimated for the Canary Islands is clearly higher.

The most important difference corresponds to fats, which according to the records represent almost 150 g of daily intake per person, while estimates derived from questionnaires would place this intake at around half. In addition to the possibility of underestimation in consumption claims of oils and other fats in the questionnaires, it is likely that the consumption estimated from data on production and trade are also biased by the different consumption patterns of tourists [21]. In fact, as Table 5 shows, according to these records oils and fats have a relative weight in the caloric intake much higher than that observed in the questionnaires.

**Table 5:** Contribution of food groups to caloric intake (%)

	NFC^a^ 2013–2017	CDC^b^ 2000–2003	ENCA^c^ 1997–1998
Edible tubers, legumes, vegetables and fruits	11.72	22.70	15.80
Cereals and preparations of cereals	21.32	12.90	21.20
Milk, cheese and other dairy products	14.89	25.70	18.40
Eggs	1.87	1.10	3.60
Meat and preparations of meat	10.96	11.95	10.90
Fisheries and preparations of fisheries	6.08	2.60	3.20
Oils and fats	12.48	7.50	5.10
Sugar, cocoa preparations and sugar confectionery	4.44	9.40	15.10
Miscellany	7.44	0.90	3.20
Non alcoholic beverages	2.36	1.70	1.50
Alcoholic beverages	6.45	2.60	2.20
Total kilocalories per person and day	3038.90	1965.36	1834.43

^a^ Compiled by authors based on the official statistics on production and external trade for the five-year period 2013–2017

^b^ CDC 2000–2003 (compiled by the authors based on (7))

^c^ ENCA 1997–1998 (compiled by the authors based on (4) and (7))

**Fig. 3:**
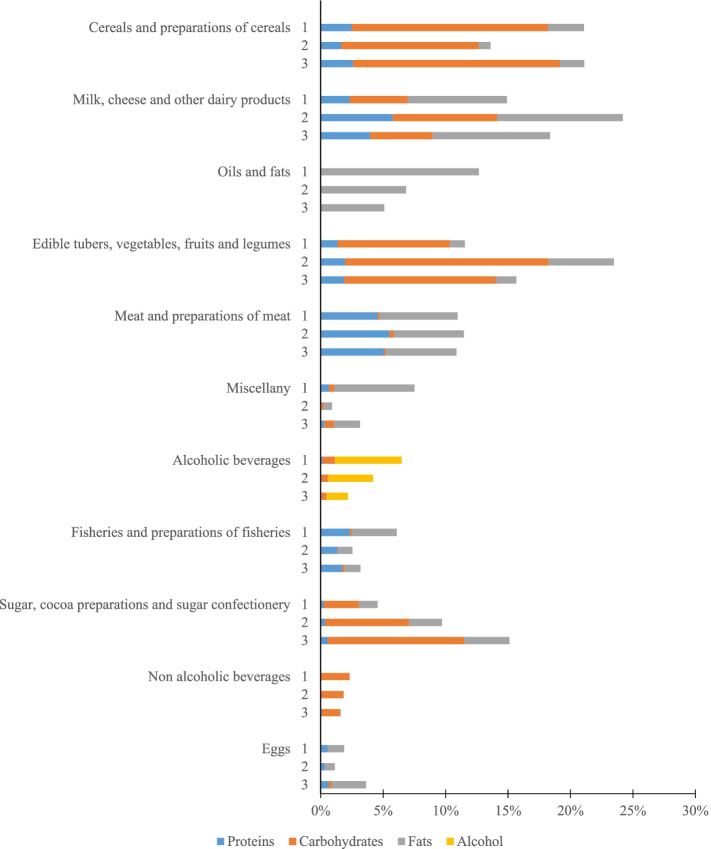
Contribution to calorie content of food groups according to nutrient input (NFC) (%). Note: 1. NFC; 2. CDC; 3. ENCA. Source: Prepared by authors based on official statistics on local production and external trade for the five-year period 2013–2017 and information obtained from the CDC 2000–2003 study (7) and the ENCA 1997–1998 study (4,7)

Table 5 shows the change in the relative weight of the caloric intake of some of the groups with the greatest contribution. Both in the case of tubers, vegetables, fruits and vegetables and in the group of dairy products, the estimates derived from the questionnaires point to a rather disparate caloric contribution, and, in any case, higher than that derived from data on production and trade. In addition, in the group of cereals and derivatives, the discrepancies between the CDC study and the ENCA study are maintained, but the latter offers a result very similar to that derived from the NFC approach. In the case of meats the contribution is quite similar in the three studies considered, while for fish the caloric contribution is higher according to the NFC approach. This is also the case with alcoholic beverages, where it seems likely that the results of the NFC approach are influenced by the effect of the tourist population, which tends to increase average alcohol consumption, as well as by a possible downward bias in public health surveys of the resident population, which tend to under-report the consumption of products considered harmful to health. Figure 3 shows the relative contributions of the caloric intake of each food group according to their contributions in protein, carbohydrate, fat and alcohol for each of the three studies compared, although these comparisons should be made with caution due to the limitations described above.

These results on diet composition are due to several factors. Despite the regional specialization in export crops, unprocessed plants show the highest self-sufficiency rates. Nevertheless, processed imported food items represent a large share of food consumption in the Canary Islands [16]. Local production specialization aimed at vegetable exports is gradually disappearing, thus forcing producers to turn to the local market. The local food industry is specialized in simple transformation processes, basically of imported inputs. Thus, if a more restrictive criterion is applied to define food industry as local only when based on local inputs, self-sufficiency rates are low. Regarding livestock production, in the Canary Islands there are strong limitations to its expansion and significant competitive disadvantages compared to imported production. Moreover, apart from a few exceptions, such as wine and goat cheese, the local food industry is supplied with raw materials of imported origin, cheaper than the local equivalents. Surprisingly even the potential of the Canary Islands-Saharan fishing grounds does not translate into catches destined for the local market of sufficient magnitude to convert this group of foods into a significant part of consumption, which barely reaches 50 g of edible weight per person per day and almost all of which corresponds to imported production.

From the perspective of demand, it is important to bear in mind that the average consumption pattern derived from the calculations does not allow the differences between socioeconomic strata to be estimated. It is also necessary to consider that the participation of the tourist population probably accentuates the relative weight of certain items.

Both supply and demand are conditioned by an institutional framework, which supports certain local productions and subsidises the import of others. It is precisely the composition of the diet, the share of imported production in it, and especially its contribution in terms of saturated fats and sugars, that requires some reflection in terms of the effects that some of these institutional measures have on diet and, finally, on health [17]. The policy implications of the results point to the importance of the links between public health problems (like obesity, diabetes), trade policy (like the SSR import regime) and subsidies for local food production. Therefore, optimisation of economic policy design under public health criteria should include a review of price-subsidy mechanisms that increase the consumption of nutrients such as sugar and saturated fats, already overrepresented in the diet, in detriment of fresh proximity food items.

## Conclusions

The approach based on records of marketed production (NFC approach) suggests that the average diet in the Canary Islands, at a cost of about eight euros per person per day, provides a hypercaloric intake of about three thousand kilocalories per day and excessive consumption of sugars and saturated fats. This approximation to the diet of the population of any age that resides in or visits the Canary Islands cannot be considered equivalent to that estimated from questionnaires applied to samples of the population resident in certain age segments. However, even though procedures for estimating the intake from official data rather than individual responses constitute an additional source of discrepancy between some results and others, this study provides a complementary view that may be useful to detect and correct biases in one or another approach, both as regards the total intake and the participation of certain food groups. Of course, the NFC approach requires an integrated statistical system about food. However, it may be of particular interest in many territories where survey-based approaches are too costly or difficult to implement. The NFC approach also offers the possibility of valuing the contributions of foods of local or imported origin, contributions that reflect the interaction ofsupply, demand and the institutional framework.

## Supplementary Information

**Additional file 1.** Nutritional equivalents per 100 grams of edible portion.

**Additional file 2.** Waste rates at the final stage of consumption (% of purchase volume)

**Additional file 3.** Average diet composition in the Canary Islands.

## Data Availability

The datasets used during the current study are available from the corresponding author on reasonable request. Data generated during this study are included in this published article and its supplementary information files.

## Footnotes

1 The case under study, the archipelago of the Canary Islands, has some particularities that should be made explicit. It is an Autonomous Region of Spain, made up of eight islands, located on the western coast of the African continent and more than 1500 km from the nearest Spanish mainland port. With a population of over 2 million inhabitants and 15 million tourists a year, it is a region of high population density with a considerable proportion of non-resident consumption. As a Spanish region, it is part of the European Union’s Single Market and Schengen Area, but it has a specific integration regime (Programme of Specific Options for the Distance and Insularity of the Canary Islands, POSEI) as an Outermost Region of the EU that includes, among others, agri-food aspects that affect both its foreign trade and support for primary production. It should be added that its status as an island region has implications for food security, which receive specific attention from the United Nations (Small Island Developing States; http://unohrlls.org/about-sids/; details in points 59–63 of the Samoa Pathway declaration: https://unohrlls.org/custom-content/uploads/2015/01/SAMOA-Pathway.pdf).

2 In option A, the production of the local food industry is identified according to the criteria of the National Accounts (location of the producing company). Option B limits the production of the local food industry to foods whose basic raw material is mainly local (water, oil, wine and cheese).

3 Specifically, we have had access to the results published in [7] and corresponding to the CDC study for the first 5364 individuals between 18 and 75 years of age enrolled in a sample that began in 2000. These individuals were given a questionnaire about the previous year and a 24-h reminder (about the previous day) 3 years after the first questionnaire (2003). On the other hand, based on the publications of [4, 7], the results corresponding to the ENCA were obtained for a sample of 1747 individuals between the ages of 6 and 75 for the period 1997–1998. A questionnaire on quantity and frequency of consumption was given to these individuals, as well as a reminder questionnaire on the previous day’s consumption on two non-consecutive days selected over a fortnightly period.

4 Other factors to be taken into account are the counting criteria used in surveys for measuring the consumption of soft drinks (including tap water consumption), the estimation of the part of fat involved in the preparation of meals but not eaten, as well as the possible underestimation of the consumption of certain products outside the home (e.g. fresh fish).
