# Supplementary material for: Food consumption in the Canary Islands: nutritional implications of food imports and local production
Source: BMC Public Health. 2022 Feb 27;22:404. doi: 10.1186/s12889-022-12805-w (PMC8882283; doi:10.1186/s12889-022-12805-w)
Supplement: Supplementary file 1 — Additional file 1. Nutritional equivalents per 100 grams of edible portion. [file 12889_2022_12805_MOESM1_ESM.docx]

**Nutritional equivalents per 100 grams of edible portion**

| **Edible tubers** | | | | | | |
| --- | --- | --- | --- | --- | --- | --- |
| Product | Energy (kcal) | Proteins (g) | Fat (g) | Carbohydrates (g) | Alcohol (g) | Saturated fatty acids (g) |
| Potato (fresh or chilled) | 73 | 2.20 | 0.20 | 15.2 | 0 | 0.03 |
| Potato (frozen) | 73 | 2.20 | 0.20 | 15.2 | 0 | 0.03 |
| Potato (dried) | 340 | 4.36 | 17.90 | 42.5 | 0 | 0 |
| Other edible tubers | 101 | 1.20 | 0.30 | 23 | 0 | 0 |

| **Vegetables** | | | | | | | | | | |
| --- | --- | --- | --- | --- | --- | --- | --- | --- | --- | --- |
| Product | Energy (kcal) | Proteins (g) | | Fat (g) | | Carbohydrates (g) | | | Alcohol (g) | Saturated fatty acids (g) |
| **Leaf and stalk vegetables** | | | | | | | | | | |
| Cabbage | 36 | | 4.30 | | 0.90 | | 2.54 | 0 | | 0.10 |
| Asparagus | 25 | | 2.90 | | 0.60 | | 2.00 | 0 | | 0.10 |
| Celery | 11 | | 0.90 | | 0.10 | | 1.50 | 0 | | 0.00 |
| Lettuce and chicory | 16 | | 1.13 | | 0.60 | | 1.40 | 0 | | 0.12 |
| Spinach | 22 | | 2.71 | | 0.80 | | 1.00 | 0 | | 0.24 |
| Other leaf and stalk vegetables | 21 | | 2.10 | | 0.20 | | 2.70 | 0 | | 0.00 |
| **Fruit vegetables** | | | | | | | | | | |
| Watermelon | 20 | | 0.40 | | 0.00 | | 4.50 | 0 | | 0.10 |
| Melon (excluded watermelon) | 27 | | 0.60 | | 0.00 | | 6.00 | 0 | | 0.03 |
| Pumpkin | 32 | | 1.20 | | 0.50 | | 5.60 | 0 | | 0.26 |
| Courgette | 17 | | 1.80 | | 0.20 | | 2.00 | 0 | | 0.04 |
| Cucumber | 12 | | 0.70 | | 0.10 | | 2.00 | 0 | | 0.00 |
| Gherkin | 12 | | 0.70 | | 0.10 | | 2.00 | 0 | | 0.00 |
| Aubergine | 20 | | 0.70 | | 0.20 | | 3.80 | 0 | | 0.00 |
| Tomato | 19 | | 0.90 | | 0.10 | | 3.50 | 0 | | 0.00 |
| Pepper (capsicum) | 29 | | 1.30 | | 0.60 | | 4.50 | 0 | | 0.14 |
| Chilli pepper | 45 | | 1.87 | | 1.10 | | 6.70 | 0 | | 0.11 |
| Strawberry | 36 | | 0.70 | | 0.50 | | 7.00 | 0 | | 0.03 |
| **Flower vegetables** | | | | | | | | | | |
| Artichoke | 23 | | 2.90 | | 0.20 | | 2.26 | 0 | | 0.00 |
| Broccoli and cauliflower | 26 | | 3.00 | | 0.40 | | 2.40 | 0 | | 0.07 |
| **Root vegetables** | | | | | | | | | | |
| Garlic | 117 | | 3.94 | | 0.23 | | 24.30 | 0 | | 0.05 |
| Onion | 26 | | 1.13 | | 0.00 | | 5.30 | 0 | | 0.00 |
| Shallot | 25 | | 3.00 | | 0.53 | | 1.90 | 0 | | 0.12 |
| Leek | 24 | | 1.60 | | 0.30 | | 3.70 | 0 | | 0.00 |
| Carrot and turnip | 34 | | 0.80 | | 0.30 | | 7.00 | 0 | | 0.05 |
| Radish and beetroot | 16 | | 0.60 | | 0.30 | | 2.60 | 0 | | 0.00 |
| **Leguminous and other vegetables** | | | | | | | | | | |
| Bean, broad bean and pea | 28 | | 2.20 | | 0.40 | | 3.70 | 0 | | 0.09 |
| Mushroom | 26 | | 1.80 | | 0.30 | | 4.00 | 0 | | 0.07 |
| Other vegetables | 19 | | 1.11 | | 0.28 | | 2.99 | 0 | | 0.06 |
| **Frozen, provisionally preserved and dried vegetables** | | | | | | | | | | |
| Frozen vegetables | 62 | | 5.40 | | 0.50 | | 8.80 | 0 | | 0.10 |
| Provisionally preserved vegetables | 24 | | 1.90 | | 0.30 | | 3.40 | 0 | | 0.07 |
| Dried vegetables | 213 | | 5.00 | | 14.00 | | 23.00 | 0 | | 0.00 |

| **Dried legumes** | | | | | | | | | |
| --- | --- | --- | --- | --- | --- | --- | --- | --- | --- |
| Product | Energy (kcal) | Proteins (g) | | Fat (g) | | Carbohydrates (g) | | Alcohol (g) | Saturated fatty acids (g) |
| Bean | 242 | | 21.10 | | 1.60 | | 34.70 | 0 | 0.53 |
| Broad bean | 358 | | 26.10 | | 1.50 | | 58.30 | 0 | 0.25 |
| Lentil | 310 | | 24.77 | | 1.17 | | 48.70 | 0 | 0.17 |
| Chickpea | 336 | | 19.30 | | 6.30 | | 49.25 | 0 | 0.43 |
| Pea | 337 | | 21.60 | | 2.30 | | 56.00 | 0 | 0.77 |
| Other dried leguminous vegetables | 247 | | 23.58 | | 0.83 | | 35.11 | 0 | 0.12 |

| **Fruits** | | | | | | | | | |
| --- | --- | --- | --- | --- | --- | --- | --- | --- | --- |
| Product | Energy (kcal) | Proteins (g) | | Fat (g) | | Carbohydrates (g) | | Alcohol (g) | Saturated fatty acids (g) |
| **Citrus fruits** | | | | | | | | | |
| Orange | 38 | | 0.80 | | 0.00 | | 8.60 | 0.00 | 0.03 |
| Mandarins; clementines, wilkings and similar citrus hybrids | 40 | | 0.80 | | 0.00 | | 9.00 | 0.00 | 0.02 |
| Lemon | 8 | | 0.60 | | 0.20 | | 1.00 | 0.00 | 0.00 |
| Grapefruit (including pomelo) | 26 | | 0.70 | | 0.10 | | 5.40 | 0.00 | 0.00 |
| Limes and other citrus fruits | 8 | | 0.60 | | 0.20 | | 1.00 | 0.00 | 0.00 |
| **Summer fruits** | | | | | | | | | |
| Apple | 50 | | 0.30 | | 0.00 | | 12.00 | 0.00 | 0.20 |
| Pear | 45 | | 0.40 | | 0.00 | | 10.60 | 0.00 | 0.01 |
| Quince | 29 | | 0.30 | | 0.23 | | 6.30 | 0.00 | 0.00 |
| Other summer fruits | 53 | | 0.40 | | 0.20 | | 12.10 | 0.00 | 0.00 |
| **Stone fruits** |  | |  | |  | |  |  |  |
| Apricot | 42 | | 0.80 | | 0.00 | | 9.50 | 0.00 | 0.01 |
| Cherry | 63 | | 0.80 | | 0.50 | | 13.50 | 0.00 | 0.07 |
| Peach (including nectarine) | 43 | | 1.40 | | 0.10 | | 9.00 | 0.00 | 0.00 |
| Plum | 54 | | 0.75 | | 0.20 | | 12.00 | 0.00 | 0.00 |
| Sloe | 47 | | 0.60 | | 0.00 | | 11.00 | 0.00 | 0.02 |
| **Other fleshy fruits** | | | | | | | | | |
| Fig | 70 | | 1.20 | | 0.00 | | 16.00 | 0.00 | 0.09 |
| Avocado | 137 | | 1.50 | | 12.00 | | 0.40 | 0.00 | 1.41 |
| Banana | 89 | | 1.20 | | 0.30 | | 20.00 | 0.00 | 0.11 |
| Date | 288 | | 2.50 | | 0.50 | | 67.00 | 0.00 | 0.00 |
| Kiwi | 52 | | 1.10 | | 0.50 | | 10.60 | 0.00 | 0.10 |
| Mango | 59 | | 0.60 | | 0.20 | | 13.40 | 0.00 | 0.05 |
| Papaya | 33 | | 0.50 | | 0.00 | | 7.60 | 0.00 | 0.00 |
| Pinneapple | 49 | | 0.50 | | 0.00 | | 11.50 | 0.00 | 0.00 |
| Other fleshy fruits | 107 | | 0.90 | | 0.10 | | 25.00 | 0.00 | 0.00 |
| **Dried fruits** | | | | | | | | | |
| Almond | 589 | | 19.13 | | 45.22 | | 6.20 | 0.00 | 4.32 |
| Nut | 595 | | 14.00 | | 63.28 | | 3.30 | 0.00 | 7.42 |
| Hazelnut | 656 | | 12.01 | | 56.25 | | 10.50 | 0.00 | 4.91 |
| Chestnut | 179 | | 2.65 | | 2.52 | | 36.50 | 0.00 | 0.42 |
| Pistachio | 594 | | 17.60 | | 49.20 | | 15.70 | 0.00 | 6.21 |
| Other dried fruits | 566 | | 1.30 | | 51.45 | | 25.35 | 0.00 | 6.90 |
| **Grape** | | | | | | | | | |
| Table grape | 68 | | 0.60 | | 0.00 | | 16.10 | 0.00 | 0.05 |
| Grape for wine | 72 | | 0.60 | | 0.70 | | 15.50 | 0.00 | 0.05 |
| Dried grape | 297 | | 2.46 | | 0.50 | | 69.30 | 0.00 | 0.16 |
| **Olive** | | | | | | | | | |
| Olive | 120 | | 1.30 | | 12.50 | | 1.00 | 0.00 | 2.60 |
| **Frozen, preserved and dried fruits** | | | | | | | | | |
| Frozen, preserved and dried fruits | 70 | | 0.72 | | 0.16 | | 16.10 | 0.00 | 0.05 |

| **Cereals** | | | | | | | | | |
| --- | --- | --- | --- | --- | --- | --- | --- | --- | --- |
| Product | Energy (kcal) | Proteins (g) | | Fat (g) | | Carbohydrates (g) | | Alcohol (g) | Saturated fatty acids (g) |
| **Winter cereals** | | | | | | | | | |
| Wheat | 314 | | 11.70 | | 2.00 | | 61.00 | 0.00 | 0.37 |
| Barley | 323 | | 10.60 | | 2.10 | | 64.00 | 0.00 | 0.30 |
| Oat | 401 | | 16.90 | | 6.90 | | 66.30 | 0.00 | 1.20 |
| Rye | 408 | | 14.80 | | 2.50 | | 79.80 | 0.00 | 0.29 |
| Triticale | 314 | | 11.70 | | 2.00 | | 61.00 | 0.00 | 0.37 |
| **Spring cereals** | | | | | | | | | |
| Rice | 387 | | 7.00 | | 0.90 | | 86.00 | 0.00 | 0.21 |
| Maize (corn) | 392 | | 8.40 | | 0.92 | | 85.60 | 0.00 | 0.13 |
| Sorghum | 314 | | 11.70 | | 2.00 | | 61.00 | 0.00 | 0.37 |
| Millet | 345 | | 11.01 | | 4.20 | | 64.40 | 0.00 | 0.72 |
| Buckwheat | 314 | | 11.70 | | 2.00 | | 61.00 | 0.00 | 0.37 |
| **Other cereals** | | | | | | | | | |
| Other cereals | 383 | | 9.00 | | 11.00 | | 61.00 | 0.00 | 2.50 |

| **Meat and edible offal** | | | | | | | | | |
| --- | --- | --- | --- | --- | --- | --- | --- | --- | --- |
| Product | Energy (kcal) | Proteins (g) | | Fat (g) | | Carbohydrates (g) | | Alcohol (g) | Saturated fatty acids (g) |
| **Meat** | | | | | | | | | |
| Bovine animals, fresh, chilled or frozen | 136 | | 23.50 | | 4.50 | | 0.00 | 0.00 | 2.00 |
| Bovine animals, salted or in brine, dried or smoked | 203 | | 32.50 | | 7.92 | | 0.00 | 0.00 | 3.67 |
| Sheep, fresh, chilled or frozen | 242 | | 15.60 | | 20.10 | | 0.00 | 0.00 | 9.96 |
| Goat, fresh, chilled or frozen | 114 | | 19.30 | | 4.00 | | 0.00 | 0.00 | 1.50 |
| Sheep and goat, salted or in brine, dried or smoked | 203 | | 32.50 | | 7.92 | | 0.00 | 0.00 | 3.67 |
| Swine, fresh, chilled or frozen | 152 | | 18.00 | | 8.90 | | 0.00 | 0.00 | 3.28 |
| Swine, salted or in brine, dried or smoked | 284 | | 25.68 | | 20.15 | | 0.18 | 0.00 | 7.08 |
| Poultry, fresh, chilled or frozen | 167 | | 20.85 | | 9.25 | | 0.00 | 0.00 | 2.66 |
| Rabbit, fresh, chilled or frozen | 132 | | 20.70 | | 5.30 | | 0.00 | 0.00 | 3.03 |
| Horses, asses, mules or hinnies, fresh, chilled or frozen | 133 | | 21.40 | | 4.60 | | 0.00 | 0.00 | 1.90 |
| Horses, asses, mules or hinnies, salted or in brine, dried or smoked | 203 | | 32.50 | | 7.92 | | 0.00 | 0.00 | 3.67 |
| **Edible offal** | | | | | | | | | |
| Bovine animals, fresh, chilled or frozen | 136 | | 21.10 | | 4.02 | | 3.50 | 0.00 | 1.50 |
| Bovine animals, salted or in brine, dried or smoked | 243 | | 35.02 | | 8.49 | | 0.00 | 0.00 | 3.30 |
| Sheep, fresh, chilled or frozen | 132 | | 15.30 | | 7.80 | | 0.00 | 0.00 | 3.00 |
| Swine, fresh, chilled or frozen | 120 | | 21.40 | | 3.70 | | 0.00 | 0.00 | 1.20 |
| Swine, salted or in brine, dried or smoked | 487 | | 7.76 | | 51.27 | | 0.08 | 0.00 | 18.96 |
| Poultry, fresh, chilled or frozen | 137 | | 22.12 | | 4.70 | | 1.20 | 0.00 | 1.58 |
| Poultry, salted or in brine, dried or smoked | 315 | | 7.09 | | 31.21 | | 2.13 | 0.00 | 8.51 |
| Rabbit, fresh, chilled or frozen | 83 | | 10.00 | | 5.00 | | 0.00 | 0.00 | 1.20 |
| Horses, asses, mules or hinnies, fresh, chilled or frozen | 242 | | 15.60 | | 20.10 | | 0.00 | 0.00 | 9.96 |
| **Other meat and edible offal** | | | | | | | | | |
| Fresh, chilled or frozen | 105 | | 22.20 | | 1.60 | | 0.00 | 0.00 | 0.80 |
| Salted or in brine, dried or smoked | 203 | | 32.50 | | 7.92 | | 0.00 | 0.00 | 3.67 |

| **Milk** | | | | | | |
| --- | --- | --- | --- | --- | --- | --- |
| Product | Energy (kcal) | Proteins (g) | Fat (g) | Carbohydrates (g) | Alcohol (g) | Saturated fatty acids (g) |
| Milk | 65 | 3.06 | 3.80 | 4.70 | 0.00 | 2.30 |

| **Egg** | | | | | | |
| --- | --- | --- | --- | --- | --- | --- |
| Product | Energy (kcal) | Proteins (g) | Fat (g) | Carbohydrates (g) | Alcohol (g) | Saturated fatty acids (g) |
| Egg | 150 | 12.50 | 11.10 | 0.00 | 0.00 | 3.10 |

| **Honey** | | | | | | |
| --- | --- | --- | --- | --- | --- | --- |
| Product | Energy (kcal) | Proteins (g) | Fat (g) | Carbohydrates (g) | Alcohol (g) | Saturated fatty acids (g) |
| Honey | 315 | 0.50 | 0.00 | 76.80 | 0.00 | 0.00 |

| **Fresh, frozen, salted or in brine, dried or smoked fish** | | | | | | | | | |
| --- | --- | --- | --- | --- | --- | --- | --- | --- | --- |
| Product | Energy (kcal) | Proteins (g) | | Fat (g) | | Carbohydrates (g) | | Alcohol (g) | Saturated fatty acids (g) |
| Salmonidae, tilapia and freshwater fish | 137 | | 17.75 | | 7.50 | | 0.41 | 0.00 | 1.62 |
| Flounder, halibut, plaice, sole, turbot, other flat fish | 86 | | 16.93 | | 1.86 | | 0.13 | 0.00 | 0.45 |
| Cod, haddock, hake, toothfish | 74 | | 15.17 | | 1.20 | | 0.00 | 0.00 | 0.24 |
| Coastal fish and several demersal fish (coalfish, pollock, blue whiting, other species in the same family; sea bass, white seabream; redfish (sebastes marinus), snapper, anglerfish, kingklip; other demersal fish) | 89 | | 16.80 | | 2.14 | | 0.78 | 0.00 | 0.54 |
| Herring, sardine, anchovy, mackerel, jack fish, black kingfish, swordfish | 146 | | 17.60 | | 7.61 | | 0.13 | 0.00 | 1.78 |
| Tuna, bonito, needlefish | 119 | | 22.00 | | 3.30 | | 0.00 | 0.00 | 0.96 |
| Shark, ray, chimaera | 117 | | 19.28 | | 4.13 | | 0.40 | 0.00 | 0.74 |
| Other pelagic fish | 126 | | 20.00 | | 1.80 | | 0.00 | 0.00 | 0.79 |
| Livers and roes | 204 | | 29.00 | | 8.00 | | 1.90 | 0.00 | 1.90 |

| **Fresh or frozen crustaceans** | | | | | | |
| --- | --- | --- | --- | --- | --- | --- |
| Product | Energy (kcal) | Proteins (g) | Fat (g) | Carbohydrates (g) | Alcohol (g) | Saturated fatty acids (g) |
| Crab, spider crab, king crab, squat lobster | 125 | 19.65 | 5.13 | 0.00 | 0.00 | 0.74 |
| Lobster | 92 | 18.15 | 2.00 | 0.00 | 0.00 | 0.24 |
| Shrimp, prawn | 95 | 19.97 | 1.07 | 1.00 | 0.00 | 0.20 |
| Other crustaceans | 87 | 17.70 | 1.40 | 0.50 | 0.00 | 0.20 |
| Other, including flours, meals and pellets of crustaceans, fit for human consumption | 60 | 13.60 | 0.50 | 0.00 | 0.00 | 0.01 |

| **Fresh, frozen, smoked molluscs and other preparations** | | | | | | |
| --- | --- | --- | --- | --- | --- | --- |
| Product | Energy (kcal) | Proteins (g) | Fat (g) | Carbohydrates (g) | Alcohol (g) | Saturated fatty acids (g) |
| Abalone, whelk, periwinkle | 102 | 17.00 | 1.40 | 5.20 | 0.00 | 0.10 |
| Oyster and limpet | 64 | 8.15 | 2.21 | 2.76 | 0.00 | 0.37 |
| Mussel | 61 | 10.80 | 1.90 | 0.00 | 0.00 | 0.41 |
| Scallop | 82 | 17.20 | 0.95 | 0.75 | 0.00 | 0.27 |
| Clam and cockle | 48 | 10.70 | 1.06 | 0.00 | 0.00 | 0.20 |
| Cuttle fish, squid, octopus | 82 | 16.44 | 1.32 | 0.80 | 0.00 | 0.35 |
| Other molluscs | 86 | 16.10 | 1.40 | 2.00 | 0.00 | 0.36 |

| **Other aquatic invertebrates** | | | | | | |
| --- | --- | --- | --- | --- | --- | --- |
| Product | Energy (kcal) | Proteins (g) | Fat (g) | Carbohydrates (g) | Alcohol (g) | Saturated fatty acids (g) |
| Fresh or chilled, frozen, smoked and other preparations | 14 | 16.30 | 7.90 | 0.00 | 0.00 | 0.00 |

| **Algae** | | | | | | |
| --- | --- | --- | --- | --- | --- | --- |
| Product | Energy (kcal) | Proteins (g) | Fat (g) | Carbohydrates (g) | Alcohol (g) | Saturated fatty acids (g) |
| Algae | 215 | 16.40 | 0.90 | 44.00 | 0.00 | 0.00 |

| **Flour** | | | | | | |
| --- | --- | --- | --- | --- | --- | --- |
| Product | Energy (kcal) | Proteins (g) | Fat (g) | Carbohydrates (g) | Alcohol (g) | Saturated fatty acids (g) |
| Wheat or meslin flour | 333 | 10.00 | 0.00 | 71.50 | 0.00 | 0.00 |
| Cereal flours other than of wheat or meslin (including bran) | 336 | 10.50 | 3.97 | 63.04 | 0.00 | 0.59 |
| Wheat meal | 355 | 11.10 | 1.20 | 72.90 | 0.00 | 0.17 |
| Cereal meal other than of wheat | 73 | 1.68 | 1.13 | 14.74 | 0.00 | 0.00 |
| Flours or meal of other vegetables | 571 | 11.00 | 0.50 | 133.00 | 0.00 | 0.10 |
| Starch and wheat gluten | 355 | 11.78 | 0.59 | 77.89 | 0.00 | 0.16 |

| **Preparations of cereals, flour, meal or starch** | | | | | | |
| --- | --- | --- | --- | --- | --- | --- |
| Product | Energy (kcal) | Proteins (g) | Fat (g) | Carbohydrates (g) | Alcohol (g) | Saturated fatty acids (g) |
| Food preparations of flour, meal, starch or malt extract (including pasta) | 201 | 8.15 | 6.80 | 28.59 | 0.00 | 2.07 |
| Tapioca and substitutes therefor prepared from starch, in the form of flakes, grains or similar forms | 159 | 1.40 | 0.30 | 38.00 | 0.00 | 0.10 |
| Prepared foods obtained by the swelling or roasting of cereals or cereal products; cereals (other than maize (corn)) in grain form or in the form of flakes or other worked grains (except flour, groats and meal) | 380 | 16.00 | 1.00 | 75.00 | 0.00 | 0.30 |
| Bread, pastry, cakes, biscuits and other bakers' wares | 286 | 8.22 | 4.96 | 51.04 | 0.00 | 2.05 |

| **Preparations of vegetables, fruits or plants** | | | | | | |
| --- | --- | --- | --- | --- | --- | --- |
| Product | Energy (kcal) | Proteins (g) | Fat (g) | Carbohydrates (g) | Alcohol (g) | Saturated fatty acids (g) |
| Potatoes prepared or preserved | 68 | 1.60 | 0.30 | 14.60 | 0.00 | 0.00 |
| Beans prepared or preserved otherwise than by vinegar or acetic acid, except ready meals | 64 | 4.71 | 0.19 | 10.62 | 0.00 | 0.05 |
| Peas prepared or preserved otherwise than by vinegar or acetic acid, except ready meals | 67 | 5.60 | 0.50 | 9.70 | 0.00 | 0.15 |
| Other leguminous or vegetables (except potatoes) prepared or preserved otherwise than by vinegar or acetic acid, except ready meals | 47 | 1.39 | 2.39 | 5.66 | 0.00 | 1.34 |
| Leguminous and vegetables (except potatoes), fruits and edible parts of plants, prepared or preserved by vinegar or acetic acid | 16 | 1.00 | 0.10 | 3.80 | 0.00 | 0.00 |
| Ready meals of leguminous and vegetables | 361 | 4.00 | 4.50 | 74.80 | 0.00 | 0.00 |
| Fruits prepared or preserved | 140 | 0.84 | 0.37 | 32.57 | 0.00 | 0.04 |
| Fruit juices and vegetables juices, unfermented and not containing added spirit | 50 | 0.30 | 0.02 | 12.17 | 0.00 | 0.00 |

| **Preparations of meat, of fish or of crustaceans, molluscs or other aquatic invertebrates** | | | | | | |
| --- | --- | --- | --- | --- | --- | --- |
| Product | Energy (kcal) | Proteins (g) | Fat (g) | Carbohydrates (g) | Alcohol (g) | Saturated fatty acids (g) |
| Sausages and similar products, of meat, meat offal or blood | 348 | 18.62 | 29.46 | 2.58 | 0.00 | 11.33 |
| Extracts and juices of meat, fish or crustaceans, molluscs or other aquatic invertebrates | 165 | 0.00 | 3.00 | 7.00 | 0.00 | 0.00 |
| Fish, crustaceans, molluscs and other aquatic invertebrates, prepared or preserved | 158 | 23.09 | 6.60 | 0.59 | 0.00 | 1.04 |

| **Soup and broths** | | | | | | |
| --- | --- | --- | --- | --- | --- | --- |
| Product | Energy (kcal) | Proteins (g) | Fat (g) | Carbohydrates (g) | Alcohol (g) | Saturated fatty acids (g) |
| Soup and broths | 333 | 12.59 | 3.24 | 62.03 | 0.00 | 1.29 |

| **Dairy products** | | | | | | |
| --- | --- | --- | --- | --- | --- | --- |
| Product | Energy (kcal) | Proteins (g) | Fat (g) | Carbohydrates (g) | Alcohol (g) | Saturated fatty acids (g) |
| Milk and cream, concentrated or containing added sugar (including powdered milk) | 336 | 8.51 | 9.25 | 53.65 | 0.00 | 6.09 |
| Cheese and curd | 342 | 19.30 | 29.69 | 0.00 | 0.00 | 0.00 |
| Yogourt and other dairy products | 95 | 2.20 | 1.92 | 16.87 | 0.00 | 1.21 |
| Butter and dairy spreads | 733 | 0.70 | 82.00 | 0.50 | 0.00 | 55.13 |

| **Ice cream and edible ice** | | | | | | |
| --- | --- | --- | --- | --- | --- | --- |
| Product | Energy (kcal) | Proteins (g) | Fat (g) | Carbohydrates (g) | Alcohol (g) | Saturated fatty acids (g) |
| Ice cream and other edible ice | 211 | 3.90 | 10.80 | 24.40 | 0.00 | 7.30 |

| **Eggs not in shell and egg yolks** | | | | | | |
| --- | --- | --- | --- | --- | --- | --- |
| Product | Energy (kcal) | Proteins (g) | Fat (g) | Carbohydrates (g) | Alcohol (g) | Saturated fatty acids (g) |
| Eggs not in shell and egg yolks | 664 | 31.70 | 59.30 | 2.10 | 0.00 | 18.00 |

| **Oils and fats** | | | | | | |
| --- | --- | --- | --- | --- | --- | --- |
| Product | Energy (kcal) | Proteins (g) | Fat (g) | Carbohydrates (g) | Alcohol (g) | Saturated fatty acids (g) |
| Olive oil and its fractions | 887 | 0.00 | 99.92 | 0.00 | 0.00 | 16.40 |
| Soya-bean oil and its fractions | 888 | 0.00 | 99.99 | 0.00 | 0.00 | 14.40 |
| Groundnut oil and its fractions | 887 | 0.00 | 99.90 | 0.00 | 0.00 | 18.80 |
| Palm oil and its fractions | 888 | 0.00 | 100.00 | 0.00 | 0.00 | 49.44 |
| Coconut oil and its fractions | 888 | 0.00 | 100.00 | 0.00 | 0.00 | 84.31 |
| Rape, colza or mustard oil and fractions thereof | 888 | 0.00 | 100.00 | 0.00 | 0.00 | 6.29 |
| Sunflower-seed or safflower and fractions thereof | 887 | 0.00 | 99.90 | 0.00 | 0.00 | 10.62 |
| Other oils for human consumption | 892 | 0.00 | 100.00 | 0.00 | 0.00 | 19.47 |
| Margarine and other edible fats | 718 | 0.90 | 80.00 | 0.90 | 0.00 | 13.75 |

| **Coffee, infusions and extracts or substitutes** | | | | | | |
| --- | --- | --- | --- | --- | --- | --- |
| Product | Energy (kcal) | Proteins (g) | Fat (g) | Carbohydrates (g) | Alcohol (g) | Saturated fatty acids (g) |
| Coffee | 298 | 13.14 | 13.63 | 4.91 | 0.00 | 0.61 |
| Infusions | 207 | 18.45 | 1.86 | 3.02 | 0.00 | 0.00 |
| Extract and substitutes of coffee, tea and other herbs | 376 | 13.59 | 3.15 | 67.11 | 0.00 | 0.52 |

| **Sugar, cocoa preparations and sugar confectionery** | | | | | | |
| --- | --- | --- | --- | --- | --- | --- |
| Product | Energy (kcal) | Proteins (g) | Fat (g) | Carbohydrates (g) | Alcohol (g) | Saturated fatty acids (g) |
| Sugar | 405 | 0.00 | 0.00 | 99.54 | 0.00 | 0.00 |
| Sugar confectionery (including white chocolate), not containing cocoa | 268 | 4.63 | 0.02 | 69.95 | 0.00 | 0.01 |
| Cocoa and cocoa preparations | 472 | 6.77 | 25.17 | 55.05 | 0.00 | 5.32 |

| **Sauces, condiments, spices, vinegar and salt** | | | | | | |
| --- | --- | --- | --- | --- | --- | --- |
| Product | Energy (kcal) | Proteins (g) | Fat (g) | Carbohydrates (g) | Alcohol (g) | Saturated fatty acids (g) |
| Spices | 355 | 12.63 | 9.34 | 54.08 | 0.00 | 1.84 |
| Sauces and other condiments | 785 | 2.10 | 87.38 | 0.10 | 0.00 | 12.80 |
| Vinegar and substitutes for vinegar | 4 | 0.40 | 0.00 | 0.60 | 0.00 | 0.00 |
| Salt | 0 | 0.00 | 0.00 | 0.00 | 0.00 | 0.00 |

| **Other food preparations** | | | | | | |
| --- | --- | --- | --- | --- | --- | --- |
| Product | Energy (kcal) | Proteins (g) | Fat (g) | Carbohydrates (g) | Alcohol (g) | Saturated fatty acids (g) |
| Other food preparations | 111 | 24.78 | 1.13 | 0.00 | 0.00 | 0.25 |

| **Bottled water** | | | | | | |
| --- | --- | --- | --- | --- | --- | --- |
| Product | Energy (kcal) | Proteins (g) | Fat (g) | Carbohydrates (g) | Alcohol (g) | Saturated fatty acids (g) |
| Bottled water | 0 | 0 | 0 | 0 | 0 | 0 |

| **Other non-alcoholic beverages** | | | | | | |
| --- | --- | --- | --- | --- | --- | --- |
| Product | Energy (kcal) | Proteins (g) | Fat (g) | Carbohydrates (g) | Alcohol (g) | Saturated fatty acids (g) |
| Other non-alcoholic beverages | 44 | 0.00 | 0.00 | 10.75 | 0.00 | 0.00 |

| **Alcoholic beverages** | | | | | | |
| --- | --- | --- | --- | --- | --- | --- |
| Product | Energy (kcal) | Proteins (g) | Fat (g) | Carbohydrates (g) | Alcohol (g) | Saturated fatty acids (g) |
| Beer | 42 | 0.50 | 0.00 | 3.12 | 3.96 | 0.00 |
| Wine | 70 | 0.22 | 0.00 | 0.28 | 9.68 | 0.00 |
| Vermouth and other fermented beverages | 83 | 0.04 | 0.00 | 8.76 | 6.80 | 0.00 |
| Spirits, liqueurs and other spirituous beverages | 219 | 0.00 | 0.00 | 0.10 | 31.41 | 0.00 |
