# Supplementary material for: Food consumption in the Canary Islands: nutritional implications of food imports and local production
Source: BMC Public Health. 2022 Feb 27;22:404. doi: 10.1186/s12889-022-12805-w (PMC8882283; doi:10.1186/s12889-022-12805-w)
Supplement: Supplementary file 2 — Additional file 2. Waste rates at the final stage of consumption (% of purchase volume) [file 12889_2022_12805_MOESM2_ESM.docx]

**Waste rates at the final stage of consumption (% of purchase volume)**

| Edible tubers | 17.00 |
| --- | --- |
| Vegetables | 7.30 |
| Legumes | 11.90 |
| Fruits | 9.40 |
| Cereals | 5.90 |
| Meat and edible offal | 4.40 |
| Milk | 1.56 |
| Eggs | 6.50 |
| Honey | 12.55 |
| Fish, crustaceans, molluscs, other aquatic invertebrates, algae | 9.60 |
| Flour | 15.00 |
| Preparations of cereals, flour, meal or starch | 14.30 |
| Preparations of vegetables, fruits or plants | 12.00 |
| Preparations of meat or fish or of crustaceans, molluscs or other aquatic invertebrates | 5.40 |
| Soups and brouths | 11.00 |
| Dairy products | 8.80 |
| Ice cream and other edible ice | 6.59 |
| Eggs not in Shell and egg yolks | 6.50 |
| Oils and fats | 12.17 |
| Coffee, teas and extracts or substitutes | 99.08 |
| Sugar, cocoa preparations and sugar confectionery | 5.18 |
| Sauces, condiments, spices, vinegar and salt | 9.68 |
| Other food preparations | 10.01 |
| Bottled water | 5.20 |
| Other non-alcoholic beverages | 7.20 |
| Alcoholic beverages | 5.40 |

Source: Prepared by the authors from Quested T., Johnson H. (2009) *Household food and drink waste in the UK*. UK: Waste and Resource Action Programme; Quested T., Murphy L. (2014) *Household food and drink waste: A product focus*. UK: Waste and Resource Action Programme, and Panel on Quantification of Food Wastage in Spanish Households (Ministry of Agriculture, Fisheries, Food and Environment of the Government of Spain).

In the unprocessed vegetable and fishery products groups, the avoidable wastage rates estimated by Quested and Johnson (2009) and Quested and Murphy (2014) were applied for comparable household food groups in the United Kingdom in 2007 and 2012^^[[1]](#footnote-1)^^. These same sources were used as a reference for the meat and egg group, and also for some food groups included within the food industry group. However, in the case of milk and honey, and also in several processed food groups (meat or fish preparations; ice cream and similar products; sugar, cocoa preparations and confectionery; sauces, condiments, vinegar and salt; other food products), wastage rates were estimated from the data for 2017 included in the Panel on Quantification of Food Wastage in Spanish Households, prepared by the Ministry of Agriculture, Fisheries, Food and Environment of the Government of Spain.

In the case of coffee, teas and extracts or substitutes, the wastage rate determined in the previous report was applied to the purchased product, and it was considered that 99% of the product used to make teas was thrown away^^[[2]](#footnote-2)^^. In the case of shelled eggs and egg products, the same wastage rate was applied as for eggs. Finally, in the group of oils and fats, it has been considered that waste is more important in oils than in margarines and it has been decided to apply a rate resulting from a weighted average of the rates applied to the most important oils, on the one hand, and to margarines and other edible oils, on the other^^[[3]](#footnote-3)^^.

1. The estimate of avoidable waste excludes the inedible part of food production. [↑](#footnote-ref-1)
2. Note that in the case of coffees, teas and extracts or substitutes, nutritional equivalents per 100 grams of edible weight of the product purchased have been used. However, only a very small portion of the product used is absorbed. [↑](#footnote-ref-2)
3. For margarines and other edible oils a waste rate of 3% has been considered (Quested and Murphy, 2014). Given the habit of frying with oils in Spain, this UK rate should be adjusted to local uses. Therefore, for the most important edible oils, and taking into account the information for Spain published by the Ministry for Ecological Transition of the Spanish Government (https://www.miteco.gob.es/es/calidad-y-evaluacion-ambiental/temas/prevencion-y-gestion-residuos/flujos/domesticos/fracciones/aceites-cocina/) concerning the consumption of these oils as well as the information on waste oils according to national consumption and cooking habits, a waste rate of 16% has been estimated. [↑](#footnote-ref-3)
