# Supplementary material for: Food consumption in the Canary Islands: nutritional implications of food imports and local production
Source: BMC Public Health. 2022 Feb 27;22:404. doi: 10.1186/s12889-022-12805-w (PMC8882283; doi:10.1186/s12889-022-12805-w)
Supplement: Supplementary file 3 — Additional file 3. Average diet composition in the Canary Islands. [file 12889_2022_12805_MOESM3_ESM.docx]

**Average diet composition in the Canary Islands**

Table A.1. Net food intake (grams of edible weight per person per day) according to food groups and product origin

|  | 2012-2016 | | | | 2013-2017 | | | |
| --- | --- | --- | --- | --- | --- | --- | --- | --- |
|  | Option A (National Accounts)^1^ | | Option B (Pure local food industry)^2^ | | Option A (National Accounts)^1^ | | Option B (Pure local food industry)^2^ | |
|  | Local | Imported | Local | Imported | Local | Imported | Local | Imported |
| Edible tubers | 81.41 | 51.46 | 81.41 | 51.46 | 82.77 | 48.54 | 82.77 | 48.54 |
| Vegetables | 125.70 | 72.45 | 125.70 | 72.45 | 132.05 | 74.38 | 132.05 | 74.38 |
| Legumes | 0.25 | 5.32 | 0.25 | 5.32 | 0.25 | 5.19 | 0.25 | 5.19 |
| Fruits | 60.90 | 98.22 | 60.90 | 98.22 | 60.78 | 101.82 | 60.78 | 101.82 |
| Cereals | 0.63 | 23.17 | 0.63 | 23.17 | 0.65 | 22.60 | 0.65 | 22.60 |
| **Primary crops** | 268.90 | 250.60 | 268.90 | 250.60 | 276.51 | 252.53 | 276.51 | 252.53 |
| Fish | 4.27 | 26.72 | 4.27 | 26.72 | 3.91 | 26.85 | 3.91 | 26.85 |
| Crustaceans | 0.04 | 0.24 | 0.04 | 0.24 | 0.04 | 0.23 | 0.04 | 0.23 |
| Molluscs | 0.05 | 0.36 | 0.02 | 0.36 | 0.01 | 0.35 | 0.01 | 0.35 |
| Other fisheries | 0.00 | 0.01 | 0.00 | 0.01 | 0.00 | 0.01 | 0.00 | 0.01 |
| **Non processed fisheries** | 4.35 | 44.32 | 4.35 | 44.32 | 3.98 | 45.79 | 3.98 | 45.79 |
| Meat and edible offal | 15.23 | 103.84 | 15.23 | 103.84 | 15.60 | 106.64 | 15.60 | 106.64 |
| Milk | 3.00 | 150.30 | 3.00 | 150.30 | 2.85 | 150.34 | 2.85 | 150.34 |
| Eggs | 19.34 | 7.69 | 19.34 | 7.69 | 19.78 | 8.41 | 19.78 | 8.41 |
| Honey | 0.41 | 1.02 | 0.41 | 1.02 | 0.41 | 1.09 | 0.41 | 1.09 |
| **Non processed livestock** | 37.98 | 262.86 | 37.98 | 262.86 | 38.64 | 266.47 | 38.64 | 266.47 |
| Flour | 7.89 | 2.15 | 0.00 | 10.04 | 7.74 | 2.25 | 0.00 | 9.99 |
| Preparations of cereals, flour, meal or starch | 91.85 | 88.11 | 0.00 | 179.96 | 95.49 | 91.24 | 0.00 | 186.73 |
| Preparations of vegetables, fruits or plants | 24.83 | 106.33 | 0.00 | 131.16 | 25.95 | 101.38 | 0.00 | 127.33 |
| Preparations of meat or fish or of crustaceans, molluscs or other aquatic invertebrates | 12.45 | 76.31 | 0.00 | 88.77 | 12.71 | 77.85 | 0.00 | 90.55 |
| Soups and brouths | 0.00 | 4.56 | 0.00 | 4.56 | 0.00 | 4.71 | 0.00 | 4.71 |
| Dairy products | 106.35 | 64.18 | 17.04 | 153.49 | 103.60 | 68.06 | 16.45 | 155.21 |
| Ice cream and other edible ice | 9.86 | 10.02 | 0.00 | 19.89 | 9.55 | 10.23 | 0.00 | 19.78 |
| Eggs not in shell and egg yolks | 0.00 | 1.96 | 0.00 | 1.96 | 0.00 | 2.20 | 0.00 | 2.20 |
| Oils and fats | 0.15 | 45.91 | 0.06 | 46.00 | 0.18 | 45.13 | 0.09 | 45.21 |
| Coffe, teas and extracts or substitutes | 0.06 | 0.05 | 0.00 | 0.10 | 0.06 | 0.05 | 0.00 | 0.10 |
| Sugar, cocoa preparations and sugar confectionery | 3.00 | 28.70 | 0.00 | 31.70 | 2.89 | 29.25 | 0.00 | 32.14 |
| Sauces, condiments, spices, vinegar and salt | 1.34 | 30.78 | 0.00 | 32.11 | 1.35 | 30.68 | 0.00 | 32.04 |
| Other food preparations | 5.23 | 8.62 | 0.00 | 13.85 | 5.69 | 9.22 | 0.00 | 14.91 |
| Bottled water | 650.16 | 171.98 | 650.16 | 171.98 | 667.97 | 194.30 | 667.97 | 194.30 |
| Other non-alcoholic beverages | 154.13 | 5.10 | 0.00 | 159.23 | 157.05 | 5.83 | 0.00 | 162.89 |
| Alcoholic beverages | 133.31 | 167.07 | 12.67 | 287.71 | 138.92 | 169.01 | 12.35 | 295.59 |
| **Food industry products** | 1,200.62 | 811.82 | 679.93 | 1,332.51 | 1,229.15 | 841.39 | 696.86 | 1,373.68 |
| **Total food** | 1,511.85 | 1,369.60 | 991.16 | 1,890.29 | 1,548.27 | 1,406.18 | 1,015.99 | 1,938.47 |

^(1)^ In option A, the production of the local food industry is identified according to the criteria of the National Accounts (locations of the producing Company).

^(2)^ Option B limits the production of the local food industry to foods whose basic raw material is mainly local (water, oil, wine and cheese).

Table A.2. Net food intake (kilocalories per person per day) according to food groups and product origin

|  | 2012-2016 | | | | 2013-2017 | | | |
| --- | --- | --- | --- | --- | --- | --- | --- | --- |
|  | Option A (National Accounts)^1^ | | Option B (Pure local food industry)^2^ | | Option A (National Accounts)^1^ | | Option B (Pure local food industry)^2^ | |
|  | Local | Imported | Local | Imported | Local | Imported | Local | Imported |
| Edible tubers | 60.36 | 37.65 | 60.36 | 37.65 | 61.34 | 35.53 | 61.34 | 35.53 |
| Vegetables | 29.48 | 27.52 | 29.48 | 27.52 | 30.73 | 28.05 | 30.73 | 28.05 |
| Legumes | 0.68 | 15.97 | 0.68 | 15.97 | 0.70 | 15.62 | 0.70 | 15.62 |
| Fruits | 41.17 | 56.66 | 41.17 | 56.66 | 41.69 | 59.27 | 41.69 | 59.27 |
| Cereals | 2.45 | 88.19 | 2.45 | 88.19 | 2.54 | 86.19 | 2.54 | 86.19 |
| **Primary crops** | 134.14 | 225.99 | 134.14 | 225.99 | 137.00 | 224.66 | 137.00 | 224.66 |
| Fish | 5.42 | 28.42 | 5.42 | 28.42 | 5.00 | 28.35 | 5.00 | 28.35 |
| Crustaceans | 0.03 | 8.30 | 0.03 | 8.30 | 0.04 | 8.97 | 0.04 | 8.97 |
| Molluscs | 0.04 | 6.88 | 0.04 | 6.88 | 0.02 | 7.42 | 0.02 | 7.42 |
| Other fisheries | 0.00 | 0.00 | 0.00 | 0.00 | 0.00 | 0.00 | 0.00 | 0.00 |
| **Non processed fisheries** | 5.49 | 43.60 | 5.49 | 43.60 | 5.06 | 44.74 | 5.06 | 44.74 |
| Meat and edible offal | 23.59 | 168.64 | 23.59 | 168.64 | 24.19 | 173.88 | 24.19 | 173.88 |
| Milk | 1.95 | 97.70 | 1.95 | 97.70 | 1.85 | 97.72 | 1.85 | 97.72 |
| Eggs | 29.01 | 11.54 | 29.01 | 11.54 | 29.67 | 12.61 | 29.67 | 12.61 |
| Honey | 1.30 | 3.22 | 1.30 | 3.22 | 1.29 | 3.43 | 1.29 | 3.43 |
| **Non processed livestock** | 55.85 | 281.10 | 55.85 | 281.10 | 57.00 | 287.64 | 57.00 | 287.64 |
| Flour | 26.28 | 7.17 | 0.00 | 33.45 | 25.77 | 7.49 | 0.00 | 33.26 |
| Preparations of cereals, flour, meal or starch | 259.01 | 247.41 | 0.00 | 506.42 | 269.50 | 256.26 | 0.00 | 525.76 |
| Preparations of vegetables, fruits or plants | 14.01 | 70.47 | 0.00 | 84.49 | 14.61 | 68.59 | 0.00 | 83.20 |
| Preparations of meat or fish or of crustaceans, molluscs or other aquatic invertebrates | 41.27 | 223.51 | 0.00 | 264.78 | 41.70 | 228.25 | 0.00 | 269.96 |
| Soups and brouths | 0.00 | 15.20 | 0.00 | 15.20 | 0.00 | 15.68 | 0.00 | 15.68 |
| Dairy products | 143.16 | 165.65 | 58.29 | 250.52 | 139.07 | 172.27 | 56.25 | 255.09 |
| Ice cream and other edible ice | 20.81 | 21.15 | 0.00 | 41.96 | 20.15 | 21.58 | 0.00 | 41.73 |
| Eggs not in shell and egg yolks | 0.01 | 12.98 | 0.00 | 12.99 | 0.01 | 14.60 | 0.00 | 14.62 |
| Oils and fats | 1.15 | 384.82 | 0.00 | 385.96 | 1.41 | 377.70 | 0.00 | 379.11 |
| Coffe, teas and extracts or substitutes | 0.17 | 0.14 | 0.00 | 0.31 | 0.17 | 0.14 | 0.00 | 0.31 |
| Sugar, cocoa preparations and sugar confectionery | 11.48 | 116.68 | 0.00 | 128.15 | 11.16 | 119.13 | 0.00 | 130.29 |
| Sauces, condiments, spices, vinegar and salt | 10.49 | 181.24 | 0.00 | 191.73 | 10.63 | 182.75 | 0.00 | 193.38 |
| Other food preparations | 5.82 | 9.59 | 0.00 | 15.41 | 6.33 | 10.26 | 0.00 | 16.59 |
| Bottled water | 0.00 | 0.00 | 0.00 | 0.00 | 0.00 | 0.00 | 0.00 | 0.00 |
| Other non-alcoholic beverages | 67.82 | 2.25 | 0.00 | 70.06 | 69.10 | 2.57 | 0.00 | 71.67 |
| Alcoholic beverages | 73.41 | 118.30 | 8.85 | 182.86 | 75.59 | 120.34 | 8.63 | 187.30 |
| **Food industry products** | 674.89 | 1,576.55 | 67.14 | 2,184.29 | 685.19 | 1,597.62 | 64.88 | 2,217.94 |
| **Total food** | 870.38 | 2,127.24 | 262.63 | 2,734.99 | 884.25 | 2,154.65 | 263.94 | 2,774.97 |

^(1)^ In option A, the production of the local food industry is identified according to the criteria of the National Accounts (locations of the producing Company).

^(2)^ Option B limits the production of the local food industry to foods whose basic raw material is mainly local (water, oil, wine and cheese).

Table A.3. Net food intake (grams of fat per person per day) according to food groups and product origin

|  | 2012-2016 | | | | 2013-2017 | | | |
| --- | --- | --- | --- | --- | --- | --- | --- | --- |
|  | Option A (National Accounts)^1^ | | Option B (Pure local food industry)^2^ | | Option A (National Accounts)^1^ | | Option B (Pure local food industry)^2^ | |
|  | Local | Imported | Local | Imported | Local | Imported | Local | Imported |
| Edible tubers | 0.17 | 0.10 | 0.17 | 0.10 | 0.17 | 0.10 | 0.17 | 0.10 |
| Vegetables | 0.39 | 0.19 | 0.39 | 0.19 | 0.41 | 0.19 | 0.41 | 0.19 |
| Legumes | 0.00 | 0.15 | 0.00 | 0.15 | 0.00 | 0.15 | 0.00 | 0.15 |
| Fruits | 0.87 | 1.01 | 0.87 | 1.01 | 0.93 | 1.10 | 0.93 | 1.10 |
| Cereals | 0.01 | 0.39 | 0.01 | 0.39 | 0.01 | 0.41 | 0.01 | 0.41 |
| **Primary crops** | 1.44 | 1.84 | 1.44 | 1.84 | 1.53 | 1.95 | 1.53 | 1.95 |
| Fish | 0.20 | 1.03 | 0.20 | 1.03 | 0.19 | 1.02 | 0.19 | 1.02 |
| Crustaceans | 0.00 | 0.10 | 0.00 | 0.10 | 0.00 | 0.11 | 0.00 | 0.11 |
| Molluscs | 0.00 | 0.12 | 0.00 | 0.12 | 0.00 | 0.13 | 0.00 | 0.13 |
| Other fisheries | 0.00 | 0.00 | 0.00 | 0.00 | 0.00 | 0.00 | 0.00 | 0.00 |
| **Non processed fisheries** | 0.20 | 1.26 | 0.20 | 1.26 | 0.19 | 1.25 | 0.19 | 1.25 |
| Meat and edible offal | 1.23 | 8.97 | 1.23 | 8.97 | 1.26 | 9.28 | 1.26 | 9.28 |
| Milk | 0.11 | 5.71 | 0.11 | 5.71 | 0.11 | 5.71 | 0.11 | 5.71 |
| Eggs | 2.15 | 0.85 | 2.15 | 0.85 | 2.20 | 0.93 | 2.20 | 0.93 |
| Honey | 0.00 | 0.00 | 0.00 | 0.00 | 0.00 | 0.00 | 0.00 | 0.00 |
| **Non processed livestock** | 3.49 | 15.54 | 3.49 | 15.54 | 3.57 | 15.92 | 3.57 | 15.92 |
| Flour | 0.00 | 0.01 | 0.00 | 0.01 | 0.00 | 0.01 | 0.00 | 0.01 |
| Preparations of cereals, flour, meal or starch | 4.63 | 4.32 | 0.00 | 8.95 | 4.81 | 4.47 | 0.00 | 9.28 |
| Preparations of vegetables, fruits or plants | 0.20 | 0.70 | 0.00 | 0.90 | 0.23 | 0.72 | 0.00 | 0.94 |
| Preparations of meat or fish or of crustaceans, molluscs or other aquatic invertebrates | 3.42 | 17.41 | 0.00 | 20.83 | 3.44 | 17.79 | 0.00 | 21.23 |
| Soups and brouths | 0.00 | 0.15 | 0.00 | 0.15 | 0.00 | 0.15 | 0.00 | 0.15 |
| Dairy products | 6.77 | 11.80 | 5.06 | 13.52 | 6.56 | 12.23 | 4.88 | 13.91 |
| Ice cream and other edible ice | 1.07 | 1.08 | 0.00 | 2.15 | 1.03 | 1.10 | 0.00 | 2.14 |
| Eggs not in shell and egg yolks | 0.00 | 1.16 | 0.00 | 1.16 | 0.00 | 1.30 | 0.00 | 1.31 |
| Oils and fats | 0.13 | 43.22 | 0.00 | 43.35 | 0.16 | 42.42 | 0.00 | 42.58 |
| Coffe, teas and extracts or substitutes | 0.01 | 0.00 | 0.00 | 0.01 | 0.01 | 0.00 | 0.00 | 0.01 |
| Sugar, cocoa preparations and sugar confectionery | 0.39 | 4.46 | 0.00 | 4.85 | 0.39 | 4.59 | 0.00 | 4.98 |
| Sauces, condiments, spices, vinegar and salt | 1.17 | 19.96 | 0.00 | 21.12 | 1.18 | 20.10 | 0.00 | 21.28 |
| Other food preparations | 0.06 | 0.10 | 0.00 | 0.16 | 0.06 | 0.10 | 0.00 | 0.17 |
| Bottled water | 0.00 | 0.00 | 0.00 | 0.00 | 0.00 | 0.00 | 0.00 | 0.00 |
| Other non-alcoholic beverages | 0.00 | 0.00 | 0.00 | 0.00 | 0.00 | 0.00 | 0.00 | 0.00 |
| Alcoholic beverages | 0.00 | 0.00 | 0.00 | 0.00 | 0.00 | 0.00 | 0.00 | 0.00 |
| **Food industry products** | 17.85 | 104.37 | 5.06 | 117.16 | 17.87 | 105.00 | 4.88 | 117.98 |
| **Total food** | 22.99 | 123.00 | 10.20 | 135.79 | 23.15 | 124.12 | 10.17 | 137.11 |

^(1)^ In option A, the production of the local food industry is identified according to the criteria of the National Accounts (locations of the producing Company).

^(2)^ Option B limits the production of the local food industry to foods whose basic raw material is mainly local (water, oil, wine and cheese).

Table A.4. Net food intake (grams of saturated fatty acids per person per day) according to food groups and product origin

|  | 2012-2016 | | | | 2013-2017 | | | |
| --- | --- | --- | --- | --- | --- | --- | --- | --- |
|  | Option A (National Accounts)^1^ | | Option B (Pure local food industry)^2^ | | Option A (National Accounts)^1^ | | Option B (Pure local food industry)^2^ | |
|  | Local | Imported | Local | Imported | Local | Imported | Local | Imported |
| Edible tubers | 0.02 | 0.02 | 0.02 | 0.02 | 0.02 | 0.01 | 0.02 | 0.01 |
| Vegetables | 0.09 | 0.03 | 0.09 | 0.03 | 0.09 | 0.04 | 0.09 | 0.04 |
| Legumes | 0.00 | 0.02 | 0.00 | 0.02 | 0.00 | 0.02 | 0.00 | 0.02 |
| Fruits | 0.12 | 0.16 | 0.12 | 0.16 | 0.13 | 0.17 | 0.13 | 0.17 |
| Cereals | 0.00 | 0.09 | 0.00 | 0.09 | 0.00 | 0.09 | 0.00 | 0.09 |
| **Primary crops** | 0.24 | 0.32 | 0.24 | 0.32 | 0.25 | 0.33 | 0.25 | 0.33 |
| Fish | 0.05 | 0.23 | 0.05 | 0.23 | 0.05 | 0.23 | 0.05 | 0.23 |
| Crustaceans | 0.00 | 0.02 | 0.00 | 0.02 | 0.00 | 0.02 | 0.00 | 0.02 |
| Molluscs | 0.00 | 0.03 | 0.00 | 0.03 | 0.00 | 0.03 | 0.00 | 0.03 |
| Other fisheries | 0.00 | 0.00 | 0.00 | 0.00 | 0.00 | 0.00 | 0.00 | 0.00 |
| **Non processed fisheries** | 0.05 | 0.28 | 0.05 | 0.28 | 0.05 | 0.28 | 0.05 | 0.28 |
| Meat and edible offal | 0.41 | 3.11 | 0.41 | 3.11 | 0.42 | 3.22 | 0.42 | 3.22 |
| Milk | 0.07 | 3.45 | 0.07 | 3.45 | 0.07 | 3.45 | 0.07 | 3.45 |
| Eggs | 0.60 | 0.24 | 0.60 | 0.24 | 0.61 | 0.26 | 0.61 | 0.26 |
| Honey | 0.00 | 0.00 | 0.00 | 0.00 | 0.00 | 0.00 | 0.00 | 0.00 |
| **Non processed livestock** | 1.08 | 6.80 | 1.08 | 6.80 | 1.09 | 6.93 | 1.09 | 6.93 |
| Flour | 0.00 | 0.00 | 0.00 | 0.00 | 0.00 | 0.00 | 0.00 | 0.00 |
| Preparations of cereals, flour, meal or starch | 1.88 | 1.68 | 0.00 | 3.55 | 1.95 | 1.73 | 0.00 | 3.68 |
| Preparations of vegetables, fruits or plants | 0.11 | 0.32 | 0.00 | 0.42 | 0.12 | 0.32 | 0.00 | 0.44 |
| Preparations of meat or fish or of crustaceans, molluscs or other aquatic invertebrates | 1.30 | 6.35 | 0.00 | 7.65 | 1.30 | 6.49 | 0.00 | 7.79 |
| Soups and brouths | 0.00 | 0.06 | 0.00 | 0.06 | 0.00 | 0.06 | 0.00 | 0.06 |
| Dairy products | 1.08 | 2.07 | 0.00 | 3.15 | 1.06 | 2.16 | 0.00 | 3.21 |
| Ice cream and other edible ice | 0.72 | 0.73 | 0.00 | 1.45 | 0.70 | 0.75 | 0.00 | 1.44 |
| Eggs not in shell and egg yolks | 0.00 | 0.35 | 0.00 | 0.35 | 0.00 | 0.40 | 0.00 | 0.40 |
| Oils and fats | 0.02 | 6.60 | 0.00 | 6.62 | 0.03 | 6.30 | 0.00 | 6.33 |
| Coffe, teas and extracts or substitutes | 0.00 | 0.00 | 0.00 | 0.00 | 0.00 | 0.00 | 0.00 | 0.00 |
| Sugar, cocoa preparations and sugar confectionery | 0.08 | 0.94 | 0.00 | 1.02 | 0.08 | 0.97 | 0.00 | 1.05 |
| Sauces, condiments, spices, vinegar and salt | 0.17 | 2.92 | 0.00 | 3.09 | 0.17 | 2.94 | 0.00 | 3.12 |
| Other food preparations | 0.01 | 0.02 | 0.00 | 0.03 | 0.01 | 0.02 | 0.00 | 0.04 |
| Bottled water | 0.00 | 0.00 | 0.00 | 0.00 | 0.00 | 0.00 | 0.00 | 0.00 |
| Other non-alcoholic beverages | 0.00 | 0.00 | 0.00 | 0.00 | 0.00 | 0.00 | 0.00 | 0.00 |
| Alcoholic beverages | 0.00 | 0.00 | 0.00 | 0.00 | 0.00 | 0.00 | 0.00 | 0.00 |
| **Food industry products** | 5.37 | 22.04 | 0.00 | 27.41 | 5.42 | 22.15 | 0.00 | 27.57 |
| **Total food** | 6.73 | 29.43 | 1.36 | 34.80 | 6.81 | 29.69 | 1.39 | 35.10 |

^(1)^ In option A, the production of the local food industry is identified according to the criteria of the National Accounts (locations of the producing Company).

^(2)^ Option B limits the production of the local food industry to foods whose basic raw material is mainly local (water, oil, wine and cheese).

Table A.5. Net food intake (grams of carbohydrates per person per day) according to food groups and product origin

|  | 2012-2016 | | | | 2013-2017 | | | |
| --- | --- | --- | --- | --- | --- | --- | --- | --- |
|  | Option A (National Accounts)^1^ | | Option B (Pure local food industry)^2^ | | Option A (National Accounts)^1^ | | Option B (Pure local food industry)^2^ | |
|  | Local | Imported | Local | Imported | Local | Imported | Local | Imported |
| Edible tubers | 12.63 | 7.84 | 12.63 | 7.84 | 12.84 | 7.40 | 12.84 | 7.40 |
| Vegetables | 4.65 | 4.86 | 4.65 | 4.86 | 4.85 | 4.96 | 4.85 | 4.96 |
| Legumes | 0.10 | 2.41 | 0.10 | 2.41 | 0.10 | 2.36 | 0.10 | 2.36 |
| Fruits | 7.27 | 10.59 | 7.27 | 10.59 | 7.23 | 10.96 | 7.23 | 10.96 |
| Cereals | 0.52 | 19.00 | 0.52 | 19.00 | 0.53 | 18.50 | 0.53 | 18.50 |
| **Primary crops** | 25.17 | 44.70 | 25.17 | 44.70 | 25.56 | 44.18 | 25.56 | 44.18 |
| Fish | 0.00 | 0.12 | 0.00 | 0.12 | 0.00 | 0.11 | 0.00 | 0.11 |
| Crustaceans | 0.00 | 0.08 | 0.00 | 0.08 | 0.00 | 0.08 | 0.00 | 0.08 |
| Molluscs | 0.00 | 0.08 | 0.00 | 0.08 | 0.00 | 0.08 | 0.00 | 0.08 |
| Other fisheries | 0.00 | 0.00 | 0.00 | 0.00 | 0.00 | 0.00 | 0.00 | 0.00 |
| **Non processed fisheries** | 0.00 | 0.27 | 0.00 | 0.27 | 0.00 | 0.28 | 0.00 | 0.28 |
| Meat and edible offal | 0.02 | 0.03 | 0.02 | 0.03 | 0.02 | 0.03 | 0.02 | 0.03 |
| Milk | 0.14 | 7.06 | 0.14 | 7.06 | 0.13 | 7.07 | 0.13 | 7.07 |
| Eggs | 0.00 | 0.00 | 0.00 | 0.00 | 0.00 | 0.00 | 0.00 | 0.00 |
| Honey | 0.32 | 0.79 | 0.32 | 0.79 | 0.31 | 0.84 | 0.31 | 0.84 |
| **Non processed livestock** | 0.48 | 7.88 | 0.48 | 7.88 | 0.47 | 7.93 | 0.47 | 7.93 |
| Flour | 5.64 | 1.52 | 0.00 | 7.16 | 5.53 | 1.59 | 0.00 | 7.12 |
| Preparations of cereals, flour, meal or starch | 45.93 | 43.71 | 0.00 | 89.63 | 47.81 | 45.27 | 0.00 | 93.08 |
| Preparations of vegetables, fruits or plants | 2.90 | 14.97 | 0.00 | 17.88 | 2.99 | 14.45 | 0.00 | 17.44 |
| Preparations of meat or fish or of crustaceans, molluscs or other aquatic invertebrates | 0.30 | 1.53 | 0.00 | 1.83 | 0.30 | 1.56 | 0.00 | 1.86 |
| Soups and brouths | 0.00 | 2.83 | 0.00 | 2.83 | 0.00 | 2.92 | 0.00 | 2.92 |
| Dairy products | 15.07 | 8.10 | 0.00 | 23.17 | 14.70 | 8.54 | 0.00 | 23.25 |
| Ice cream and other edible ice | 2.41 | 2.45 | 0.00 | 4.85 | 2.33 | 2.50 | 0.00 | 4.83 |
| Eggs not in shell and egg yolks | 0.00 | 0.04 | 0.00 | 0.04 | 0.00 | 0.05 | 0.00 | 0.05 |
| Oils and fats | 0.00 | 0.12 | 0.00 | 0.12 | 0.00 | 0.12 | 0.00 | 0.12 |
| Coffe, teas and extracts or substitutes | 0.00 | 0.01 | 0.00 | 0.01 | 0.00 | 0.01 | 0.00 | 0.01 |
| Sugar, cocoa preparations and sugar confectionery | 1.92 | 18.22 | 0.00 | 20.14 | 1.84 | 18.50 | 0.00 | 20.35 |
| Sauces, condiments, spices, vinegar and salt | 0.00 | 0.41 | 0.00 | 0.41 | 0.00 | 0.45 | 0.00 | 0.45 |
| Other food preparations | 0.00 | 0.00 | 0.00 | 0.00 | 0.00 | 0.00 | 0.00 | 0.00 |
| Bottled water | 0.00 | 0.00 | 0.00 | 0.00 | 0.00 | 0.00 | 0.00 | 0.00 |
| Other non-alcoholic beverages | 16.57 | 0.55 | 0.00 | 17.12 | 16.88 | 0.63 | 0.00 | 17.51 |
| Alcoholic beverages | 3.56 | 3.56 | 0.04 | 7.09 | 3.75 | 3.66 | 0.03 | 7.37 |
| **Food industry products** | 94.29 | 98.02 | 0.04 | 192.27 | 96.13 | 100.26 | 0.03 | 196.36 |
| **Total food** | 119.95 | 150.87 | 25.69 | 245.12 | 122.16 | 152.66 | 26.06 | 248.76 |

^(1)^ In option A, the production of the local food industry is identified according to the criteria of the National Accounts (locations of the producing Company).

^(2)^ Option B limits the production of the local food industry to foods whose basic raw material is mainly local (water, oil, wine and cheese).

Table A.6. Net food intake (grams of proteins per person per day) according to food groups and product origin

|  | 2012-2016 | | | | 2013-2017 | | | |
| --- | --- | --- | --- | --- | --- | --- | --- | --- |
|  | Option A (National Accounts)^1^ | | Option B (Pure local food industry)^2^ | | Option A (National Accounts)^1^ | | Option B (Pure local food industry)^2^ | |
|  | Local | Imported | Local | Imported | Local | Imported | Local | Imported |
| Edible tubers | 1.76 | 1.13 | 1.76 | 1.13 | 1.79 | 1.06 | 1.79 | 1.06 |
| Vegetables | 1.72 | 1.52 | 1.72 | 1.52 | 1.79 | 1.54 | 1.79 | 1.54 |
| Legumes | 0.06 | 1.18 | 0.06 | 1.18 | 0.06 | 1.15 | 0.06 | 1.15 |
| Fruits | 0.60 | 0.97 | 0.60 | 0.97 | 0.60 | 1.02 | 0.60 | 1.02 |
| Cereals | 0.06 | 1.79 | 0.06 | 1.79 | 0.06 | 1.75 | 0.06 | 1.75 |
| **Primary crops** | 4.19 | 6.58 | 4.19 | 6.58 | 4.29 | 6.53 | 4.29 | 6.53 |
| Fish | 0.88 | 4.67 | 0.88 | 4.67 | 0.79 | 4.69 | 0.79 | 4.69 |
| Crustaceans | 0.01 | 1.74 | 0.01 | 1.74 | 0.01 | 1.87 | 0.01 | 1.87 |
| Molluscs | 0.01 | 1.36 | 0.01 | 1.36 | 0.00 | 1.47 | 0.00 | 1.47 |
| Other fisheries | 0.00 | 0.00 | 0.00 | 0.00 | 0.00 | 0.00 | 0.00 | 0.00 |
| **Non processed fisheries** | 0.89 | 7.77 | 0.89 | 7.77 | 0.80 | 8.04 | 0.80 | 8.04 |
| Meat and edible offal | 3.09 | 21.82 | 3.09 | 21.82 | 3.17 | 22.43 | 3.17 | 22.43 |
| Milk | 0.09 | 4.60 | 0.09 | 4.60 | 0.09 | 4.60 | 0.09 | 4.60 |
| Eggs | 2.42 | 0.96 | 2.42 | 0.96 | 2.47 | 1.05 | 2.47 | 1.05 |
| Honey | 0.00 | 0.01 | 0.00 | 0.01 | 0.00 | 0.01 | 0.00 | 0.01 |
| **Non processed livestock** | 5.60 | 27.39 | 5.60 | 27.39 | 5.73 | 28.09 | 5.73 | 28.09 |
| Flour | 0.79 | 0.22 | 0.00 | 1.01 | 0.77 | 0.23 | 0.00 | 1.00 |
| Preparations of cereals, flour, meal or starch | 7.56 | 7.80 | 0.00 | 15.36 | 7.86 | 8.09 | 0.00 | 15.95 |
| Preparations of vegetables, fruits or plants | 0.18 | 1.10 | 0.00 | 1.28 | 0.19 | 1.11 | 0.00 | 1.31 |
| Preparations of meat or fish or of crustaceans, molluscs or other aquatic invertebrates | 2.37 | 15.20 | 0.00 | 17.57 | 2.43 | 15.50 | 0.00 | 17.93 |
| Soups and brouths | 0.00 | 0.57 | 0.00 | 0.57 | 0.00 | 0.59 | 0.00 | 0.59 |
| Dairy products | 5.26 | 6.82 | 3.29 | 8.79 | 5.09 | 7.07 | 3.17 | 8.99 |
| Ice cream and other edible ice | 0.38 | 0.39 | 0.00 | 0.78 | 0.37 | 0.40 | 0.00 | 0.77 |
| Eggs not in shell and egg yolks | 0.00 | 0.62 | 0.00 | 0.62 | 0.00 | 0.70 | 0.00 | 0.70 |
| Oils and fats | 0.00 | 0.12 | 0.00 | 0.12 | 0.00 | 0.12 | 0.00 | 0.12 |
| Coffe, teas and extracts or substitutes | 0.01 | 0.01 | 0.00 | 0.01 | 0.01 | 0.01 | 0.00 | 0.01 |
| Sugar, cocoa preparations and sugar confectionery | 0.16 | 1.59 | 0.00 | 1.75 | 0.16 | 1.63 | 0.00 | 1.79 |
| Sauces, condiments, spices, vinegar and salt | 0.03 | 0.58 | 0.00 | 0.60 | 0.03 | 0.59 | 0.00 | 0.62 |
| Other food preparations | 1.30 | 2.14 | 0.00 | 3.43 | 1.41 | 2.28 | 0.00 | 3.69 |
| Bottled water | 0.00 | 0.00 | 0.00 | 0.00 | 0.00 | 0.00 | 0.00 | 0.00 |
| Other non-alcoholic beverages | 0.00 | 0.00 | 0.00 | 0.00 | 0.00 | 0.00 | 0.00 | 0.00 |
| Alcoholic beverages | 0.59 | 0.55 | 0.03 | 1.11 | 0.62 | 0.55 | 0.03 | 1.14 |
| **Food industry products** | 18.62 | 37.69 | 3.32 | 53.00 | 18.94 | 38.87 | 3.20 | 54.61 |
| **Total food** | 29.29 | 79.44 | 13.99 | 94.74 | 29.77 | 81.52 | 14.03 | 97.27 |

^(1)^ In option A, the production of the local food industry is identified according to the criteria of the National Accounts (locations of the producing Company).

^(2)^ Option B limits the production of the local food industry to foods whose basic raw material is mainly local (water, oil, wine and cheese).

Table A.7. Net food intake (grams of alcohol per person per day) according to food groups and product origin

|  | 2012-2016 | | | | 2013-2017 | | | |
| --- | --- | --- | --- | --- | --- | --- | --- | --- |
|  | Option A (National Accounts)^1^ | | Option B (Pure local food industry)^2^ | | Option A (National Accounts)^1^ | | Option B (Pure local food industry)^2^ | |
|  | Local | Imported | Local | Imported | Local | Imported | Local | Imported |
| Beer | 4.47 | 3.30 | 0.00 | 7.77 | 4.70 | 3.30 | 0.00 | 8.00 |
| Wine | 1.23 | 5.70 | 1.23 | 5.70 | 1.20 | 5.76 | 1.20 | 5.76 |
| Vermouth and other fermented beverages | 0.00 | 0.60 | 0.00 | 0.60 | 0.00 | 0.68 | 0.00 | 0.68 |
| Spirits, liqueurs and other spirituous beverages | 2.47 | 4.99 | 0.00 | 7.47 | 2.45 | 5.09 | 0.00 | 7.55 |
| **Alcoholic beverages** | 8.16 | 14.60 | 1.23 | 21.53 | 8.35 | 14.83 | 1.20 | 21.99 |
| **Total food** | 8.16 | 14.60 | 1.23 | 21.53 | 8.35 | 14.83 | 1.20 | 21.99 |

^(1)^ In option A, the production of the local food industry is identified according to the criteria of the National Accounts (locations of the producing Company).

^(2)^ Option B limits the production of the local food industry to foods whose basic raw material is mainly local (water, oil, wine and cheese).

Table A.8. Net food intake (euros per person per day) according to food groups and product origin

|  | 2012-2016 | | | | 2013-2017 | | | |
| --- | --- | --- | --- | --- | --- | --- | --- | --- |
|  | Option A (National Accounts)^1^ | | Option B (Pure local food industry)^2^ | | Option A (National Accounts)^1^ | | Option B (Pure local food industry)^2^ | |
|  | Local | Imported | Local | Imported | Local | Imported | Local | Imported |
| Edible tubers | 0.12 | 0.07 | 0.12 | 0.07 | 0.12 | 0.07 | 0.12 | 0.07 |
| Vegetables | 0.39 | 0.19 | 0.39 | 0.19 | 0.41 | 0.19 | 0.41 | 0.19 |
| Legumes | 0.00 | 0.01 | 0.00 | 0.01 | 0.00 | 0.01 | 0.00 | 0.01 |
| Fruits | 0.21 | 0.29 | 0.21 | 0.29 | 0.22 | 0.31 | 0.22 | 0.31 |
| Cereals | 0.00 | 0.03 | 0.00 | 0.03 | 0.00 | 0.03 | 0.00 | 0.03 |
| **Primary crops** | 0.72 | 0.59 | 0.72 | 0.59 | 0.75 | 0.61 | 0.75 | 0.61 |
| Fish | 0.09 | 0.18 | 0.09 | 0.18 | 0.09 | 0.19 | 0.09 | 0.19 |
| Crustaceans | 0.00 | 0.11 | 0.00 | 0.11 | 0.00 | 0.12 | 0.00 | 0.12 |
| Molluscs | 0.00 | 0.09 | 0.00 | 0.09 | 0.00 | 0.10 | 0.00 | 0.10 |
| Other fisheries | 0.00 | 0.00 | 0.00 | 0.00 | 0.00 | 0.00 | 0.00 | 0.00 |
| **Non processed fisheries** | 0.09 | 0.38 | 0.09 | 0.38 | 0.09 | 0.41 | 0.09 | 0.41 |
| Meat and edible offal | 0.13 | 0.75 | 0.13 | 0.75 | 0.13 | 0.77 | 0.13 | 0.77 |
| Milk | 0.00 | 0.13 | 0.00 | 0.13 | 0.00 | 0.12 | 0.00 | 0.12 |
| Eggs | 0.07 | 0.03 | 0.07 | 0.03 | 0.07 | 0.03 | 0.07 | 0.03 |
| Honey | 0.00 | 0.01 | 0.00 | 0.01 | 0.00 | 0.01 | 0.00 | 0.01 |
| **Non processed livestock** | 0.20 | 0.91 | 0.20 | 0.91 | 0.21 | 0.94 | 0.21 | 0.94 |
| Flour | 0.01 | 0.00 | 0.00 | 0.01 | 0.01 | 0.00 | 0.00 | 0.01 |
| Preparations of cereals, flour, meal or starch | 0.30 | 0.31 | 0.00 | 0.61 | 0.31 | 0.32 | 0.00 | 0.63 |
| Preparations of vegetables, fruits or plants | 0.05 | 0.40 | 0.00 | 0.46 | 0.06 | 0.41 | 0.00 | 0.47 |
| Preparations of meat or fish or of crustaceans, molluscs or other aquatic invertebrates | 0.09 | 0.56 | 0.00 | 0.65 | 0.10 | 0.59 | 0.00 | 0.68 |
| Soups and brouths | 0.00 | 0.04 | 0.00 | 0.04 | 0.00 | 0.04 | 0.00 | 0.04 |
| Dairy products | 0.35 | 0.40 | 0.17 | 0.58 | 0.48 | 0.48 | 0.17 | 0.79 |
| Ice cream and other edible ice | 0.06 | 0.06 | 0.00 | 0.12 | 0.06 | 0.06 | 0.00 | 0.12 |
| Eggs not in shell and egg yolks | 0.00 | 0.01 | 0.00 | 0.01 | 0.00 | 0.01 | 0.00 | 0.01 |
| Oils and fats | 0.00 | 0.10 | 0.00 | 0.10 | 0.00 | 0.10 | 0.00 | 0.10 |
| Coffe, teas and extracts or substitutes | 0.05 | 0.06 | 0.00 | 0.11 | 0.04 | 0.06 | 0.00 | 0.11 |
| Sugar, cocoa preparations and sugar confectionery | 0.02 | 0.24 | 0.00 | 0.26 | 0.02 | 0.24 | 0.00 | 0.27 |
| Sauces, condiments, spices, vinegar and salt | 0.00 | 0.08 | 0.00 | 0.08 | 0.00 | 0.08 | 0.00 | 0.09 |
| Other food preparations | 0.03 | 0.04 | 0.00 | 0.07 | 0.03 | 0.05 | 0.00 | 0.08 |
| Bottled water | 0.25 | 0.07 | 0.25 | 0.07 | 0.25 | 0.07 | 0.25 | 0.07 |
| Other non-alcoholic beverages | 0.10 | 0.00 | 0.00 | 0.10 | 0.10 | 0.00 | 0.00 | 0.10 |
| Alcoholic beverages | 0.33 | 0.63 | 0.07 | 0.89 | 0.33 | 0.60 | 0.06 | 0.87 |
| **Food industry products** | 1.65 | 3.01 | 0.49 | 4.17 | 1.80 | 3.12 | 0.48 | 4.44 |
| **Total food** | 2.67 | 4.89 | 1.51 | 6.05 | 2.85 | 5.08 | 1.53 | 6.39 |

^(1)^ In option A, the production of the local food industry is identified according to the criteria of the National Accounts (locations of the producing Company).

^(2)^ Option B limits the production of the local food industry to foods whose basic raw material is mainly local (water, oil, wine and cheese).
